# Profit efficiency of smallholder dairy production systems: Empirical evidence from Northern Uganda

**DOI:** 10.1371/journal.pone.0333337

**Published:** 2026-08-24

**Authors:** Peter Okello Oyoo, Simon Okwera, Daniel Micheal Okello, Basil Mugonola

**Affiliations:** 1 Department of Rural Development and Agribusiness, Faculty of Agriculture and Environment, Gulu University, Gulu, Uganda; 2 Department of Agribusiness and Natural Resource Economics, Faculty of Agriculture and Environmental Sciences, Kabale University, Kabale, Uganda; Federal University of Agriculture and Development Studies, Iragbiji (FUADSI), Nigeria, NIGERIA

## Abstract

This study examined the profit efficiency of smallholder dairy farmers operating under different dairy production systems in Gulu District and Gulu City. Specifically, the study assessed the determinants of profit efficiency among farmers practicing zero-grazing, tethering, and herding dairy production systems. A cross-sectional survey design was employed using quantitative primary data collected from 191 smallholder dairy households selected through a multistage sampling technique. The stochastic profit frontier model based on the Cobb-Douglas functional specification was used to estimate profit efficiency and identify factors influencing inefficiency across the dairy production systems. The findings revealed considerable variation in profit efficiency determinants across the three dairy production systems. For the overall dairy production system, equipment costs positively influenced profit efficiency (β = 0.069, p < 0.05), while feed costs negatively affected profitability (β = −0.145, p < 0.01). Under the zero-grazing system, herd size significantly improved profit efficiency (β = 0.700, p < 0.01), whereas feed costs reduced profitability (β = −0.553, p < 0.01). Within the tethering system, land size (β = 0.343, p < 0.05) and feed costs (β = 0.235, p < 0.01) positively influenced profit efficiency. In contrast, under the herding system, land size negatively affected profit efficiency (β = −0.482, p < 0.01), while equipment costs positively influenced profitability (β = 0.313, p < 0.05). The inefficiency model showed that education level, improved breed types, and market proximity significantly reduced inefficiency across the overall dairy production systems. The estimated gamma value (γ = 0.93454, p < 0.001) indicated that about 93.45% of the variation in dairy profit performance was attributed to inefficiency effects. The overall mean profit efficiency score was 0.6121, implying that farmers could improve profit efficiency by 26.14% if they operated at the level of the most efficient farmers. Among the production systems, the zero-grazing system recorded the highest mean profit efficiency score (0.9886), followed by the herding system (0.5163), while the tethering system had the lowest efficiency score (0.3572). The study concludes that improving feed management, strengthening extension services, promoting farmer education, and increasing investment in productive dairy assets are essential for improving profit efficiency among smallholder dairy farmers.

## 1. Introduction

The global livestock sector plays a critical role in supporting livelihoods, food security, and rural economic development, particularly in developing countries where livestock production remains a major source of income and nutrition. Dairy production, in particular, contributes significantly to household welfare through the provision of milk, employment opportunities, and income generation for millions of smallholder farmers worldwide. According to recent studies, the dairy sector supports the livelihoods of nearly one billion people globally and remains an important pathway for poverty reduction and nutritional improvement among rural households [1–3]. In Sub-Saharan Africa, dairy farming has increasingly become an important component of agricultural transformation due to rising urbanization, growing demand for animal protein, and expanding regional dairy markets [4–6].

In Uganda, the dairy sector is among the fastest-growing agricultural sub-sectors and contributes significantly to household income, employment creation, and national export earnings. The country is currently one of the leading dairy exporters within the East African Community, with dairy commercialization continuing to expand across both rural and peri-urban areas [7]. The adoption of improved dairy breeds and modern dairy production technologies has further enhanced milk productivity, market participation, and household welfare among smallholder farmers [8]. Existing evidence indicates that improved dairy technologies contribute to increased milk yields, higher household income, and poverty reduction, particularly among smallholder farmers with limited land holdings who rely heavily on dairy production for their livelihoods [8,9]. In addition, dairy products have increasingly become an important source of dietary protein and nutrition among Ugandan households [10].

The growth of Uganda’s dairy industry has largely been driven by expansion in dairy infrastructure, improved milk marketing systems, and increased private sector investment within the dairy value chain [11]. The establishment of milk collection centers and dairy cooperatives has strengthened milk aggregation, storage, and market accessibility, thereby enhancing commercialization opportunities for smallholder dairy farmers [12–14]. Despite these developments, milk productivity and profitability among smallholder dairy farmers remain relatively low and highly variable across production systems and regions. Such variations are associated with differences in feeding practices, breed quality, land availability, access to veterinary services, extension support, and farm management practices [15,16].

Smallholder dairy farmers in Uganda predominantly operate under three major production systems, namely zero-grazing, tethering, and herding systems, each characterized by distinct management practices and resource requirements. The zero-grazing system involves confinement of dairy animals with controlled feeding and close monitoring of animal health and productivity. This system is often associated with improved feed management, efficient resource utilization, and relatively higher milk productivity [17]. However, zero-grazing systems require substantial investment in housing structures, feed storage facilities, and water infrastructure, which may limit adoption among resource-constrained smallholder farmers [18]. Tethering systems allow farmers to control grazing areas and feed intake through restrained grazing management, potentially improving feed utilization and animal nutrition [19]. Herding systems, on the other hand, are largely extensive and depend on communal grazing lands, making them highly vulnerable to seasonal feed shortages, climate variability, and declining pasture quality [20].

The dairy sector continues to face several production and marketing challenges that constrain productivity and profit efficiency among smallholder farmers. Climate change, declining pasture quality, water scarcity, animal diseases, and rising feed costs increasingly threaten sustainable dairy production systems across Africa [21,22]. Environmental degradation arising from deforestation, overgrazing, and poor land management practices has further contributed to declining grazing resources and reduced livestock productivity [23]. In addition, fluctuating milk prices, limited access to credit, weak rural infrastructure, and high transaction costs continue to reduce profitability among smallholder dairy farmers [24]. These constraints are particularly severe in Northern Uganda, especially within the Acholi sub-region, where dairy production remains largely dominated by traditional and low-input production systems.

Improving efficiency in dairy production has therefore become increasingly important for enhancing farm profitability, household welfare, and sustainable agricultural transformation. Efficient dairy production systems can improve milk output, reduce production costs, and strengthen household food and nutritional security through increased availability of dairy products such as milk, yoghurt, and cheese [25,26]. Studies have shown that improved feeding strategies, effective breeding practices, access to veterinary services, and adoption of modern dairy technologies can substantially enhance dairy productivity and farm profitability [27–29].

Despite the growing importance of dairy farming in Uganda, empirical evidence on profit efficiency across different smallholder dairy production systems remains limited, particularly in Northern Uganda where dairy commercialization is still emerging. Most existing studies known to the authors have focused on milk production, adoption of dairy technologies, or general livestock productivity with limited attention to comparative profit efficiency across zero-grazing, tethering, and herding systems. Furthermore, little is known about how farm-specific, socioeconomic, and institutional factors influence profit efficiency within these production systems. This study, therefore, sought to examine the profit efficiency of smallholder dairy farmers under different dairy production systems in Northern Uganda and identify the factors influencing inefficiency across the production systems.

### 1.1. Conceptual framework

**Fig 1:**
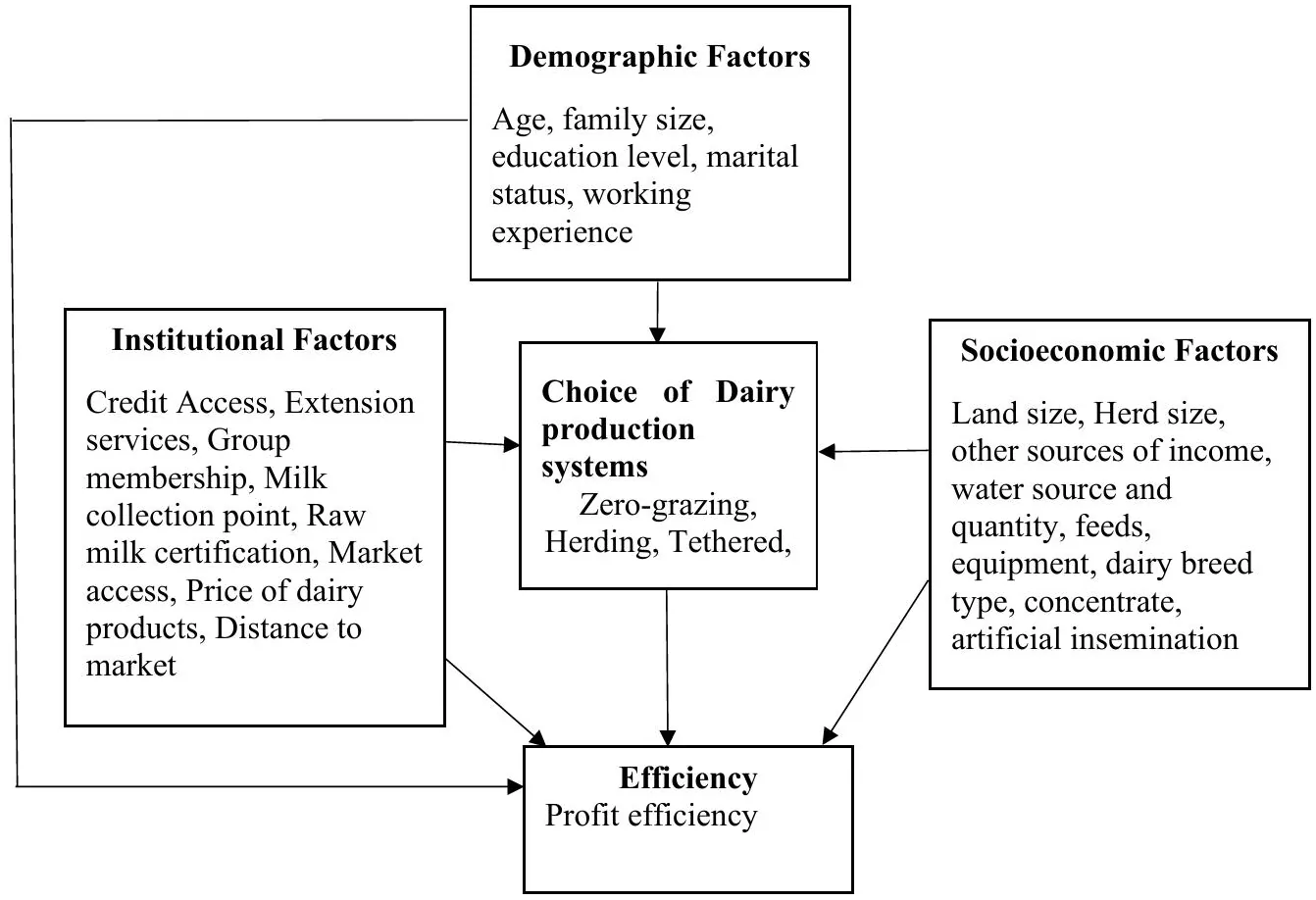
Conceptual framework shows factors influencing dairy production system choice and profit efficiency.

The framework presented in Fig 1. illustrates the interrelationship among the various factors influencing the technical, allocative, and profit efficiency of smallholder dairy production systems. It demonstrates how farm-specific, socioeconomic, and institutional variables interact to shape the efficiency performance of dairy farmers across the different production systems.

Demographic factors (age, family size, education level, marital status, and working experience) shape the farmer’s choice of dairy production system (zero-grazing, tethering, or herding). Institutional factors (credit access, extension services, group membership, milk collection point, raw milk certification, market access, price of dairy products, and distance to market) and socioeconomic factors (land size, herd size, other sources of income, water source and quantity, feeds, equipment, dairy breed type, concentrate use, and artificial insemination) influence both the choice of production system and profit efficiency directly.

## 2. Materials and methods

### 2.1. Study area

The study was conducted in northern Uganda in the Gulu district and Gulu City. Gulu City represents an urban setting with strong market demand and infrastructure, while the district reflect rural contexts where external support programs significantly influence dairy farming practices. Together, they provide a comprehensive view of the dynamics shaping smallholder dairy production in Northern Uganda. According to the Uganda Bureau of Statistics (UBOS) 2021 National Livestock Census, the Northern region had about 1,372,626 livestock-keeping households constituting 20.2% of the national livestock-keeping households with about 2.4 million cattle representing 17% of the total cattle in Uganda [30]**.** Gulu District and Gulu City are in the Acholi Sub-region. Acholi sub-region has an average cattle production with about 159,560 cattle-keeping households and an average herd size of 5.2 [30]**.** Geographically, Gulu City is located at approximately 2°46′54N and 32°17′57E, at an elevation of about 1,100 meters above sea level. The city covers an area of 1,721 km² and has a population of approximately 233,271 people, resulting in a population density of 135.5 persons per square kilometer. Gulu District is situated at approximately 2°45N and 32°00E and lies at a similar elevation of about 1,100 meters above sea level. The district covers a larger geographical area of 3,452.1 km² and has a population of about 396,500 people, with a population density of 114.9 persons per square kilometer.

### 2.2. Study design, sampling, and data collection

This study employed a cross-sectional survey design, drawing on quantitative primary data collected from smallholder dairy farmers in Gulu District and Gulu City, Northern Uganda. These locations were purposively selected as they represent the broader smallholder dairy production systems in the region, characterized by distinctive agroecological conditions, producer structures, land coverage, and production potential.

A multi-stage sampling technique was applied. In the first stage, three sub-counties from Gulu District (Awach, Paicho, and Patiko) and two divisions from Gulu City (Laroo-Pece and Bar-dege-Layibi) were randomly selected from the 11 sub-counties and four divisions, respectively. In the second stage, households engaged in dairy farming were identified with the assistance of agricultural extension workers. From these, households practicing the three predominant production systems, namely zero-grazing, tethering, and herding were purposively included to ensure a homogeneous group of respondents for comparative analysis.

The study population consisted of smallholder dairy farmers in the select sub-counties that registered with the District/City Veterinary Officer. At the time of the study, there were 380 registered smallholder dairy farmers distributed across the three sub-counties of Gulu district and two divisions of Gulu City (Table 1). Given this population, the sample size was determined following Krejcie and Morgan’s formula for sample size determination [31], which follows from equation (1).

**Table 1:** Distribution of smallholder dairy farmers per sub-county.

Sub-counties/Division	Smallholder dairy farmers	Proportion	Sample size
Awach	75	0.20	38
Bar-dege-layibi	50	0.13	25
Laroo-pece	55	0.14	28
Paicho	80	0.21	40
Patiko	120	0.32	60
**Total**	**380**		**191**


$$\begin{array}{c} s=\frac{\chi^{\text{2}}NP(\text{1}-P)}{d^{\text{2}}(N-\text{1})+\chi^{\text{2}}P(\text{1}-P)} \end{array}$$ (1)

Where: s is the required sample size, $\chi^{\text{2}}\;$is the table value of chi-square value for one degree of freedom at the desired (95%) confidence level (3.841), N is the population size, P is the population proportion (assumed to be 0.50 since this would provide maximum sample size), and d is the degree of accuracy expressed as a proportion (0.05). Substituting these parameters in the formula gives:


$$\begin{array}{c} s=\frac{\text{3}.\text{8}\text{4}\text{1}*\text{3}\text{8}\text{0}*\text{0}.\text{5}(\text{1}-\text{0}.\text{5}\text{0})}{{\text{0}.\text{0}\text{5}}^{\text{2}}(\text{3}\text{8}\text{0}-\text{1})+\text{3}.\text{8}\text{4}\text{1}*\text{0}.\text{5}\text{0}(\text{1}-\text{0}.\text{5}\text{0})}=\text{191}.\text{2}\approx \text{191} \end{array}$$

Thus, from the Krejcie and Morgan’s formula, the appropriate sample size corresponding to a population of 380 is 191. Consequently, the study used a sample size of 191, distributed proportionately across the study location as shown in Table 1. This ensured that the distribution of respondents reflected the relative size of the dairy farming population in each sub-county or division.

Data collection relied on a structured questionnaire, designed to generate quantitative insights into farmers’ demographic and socioeconomic characteristics, production practices, and constraints. A face-to-face interview using a structured questionnaire was selected due to its capacity to produce standardized, comparable responses, thereby enhancing the reliability and validity of the findings. These interviews were conducted with smallholder dairy farmers in their respective households in the study area with the consent of the local authorities, through an approved introductory letter.

The protocol for this study was reviewed and approved by the Gulu University Research Ethics Committee (GUREC) under application number GUREC-2023–525. Data collection was carried out from 20/06/2023–30/09/2023. Prior to data collection, written informed consent was obtained from all participants after explaining the purpose, procedures, potential risks, and benefits of the study.

Furthermore, the study participants were informed that they could leave the interview at any time, and their information would be kept confidential. To ensure confidentiality, all respondents were given a secret code to protect their person identity. Respondents voluntarily signed an informed consent form before participating in the survey and interviews. Additionally, the study excluded minors since they did not meet the inclusion criteria. The inclusion criteria of this study included dairy farmers who were aged 18 years and above, residing in the study area.

### 2.3. Data analysis

Collected data were coded and entered in STATA and SPSS statistical packages. After entry, the data were cleaned for potential outliers before subjecting them to rigorous statistical analysis. The data cleaning process involved conducting preliminary descriptive analysis to identify irregularities and inconsistencies in data entry, which were corrected by cross-checking the questionnaires. Outliers were also identified using the scatter plot, examined, and dealt with using robust techniques. The analyzed data are presented in the form of tables. The study used a categorical chi-square test and a one-way ANOVA test to categorize and compare dairy farmers’ production systems (Zero-grazing, Tethered, and herding) characteristics. The study also used stochastic frontier approach to assess profit efficiency.

### 2.4. The empirical model for measuring profit efficiency

The study measured the profit efficiency of smallholder dairy farmers using stochastic profit frontier Farrel’s work led to the development of the stochastic frontier approach (SFA) [32]. Later, Aigner et al. [33] proposed the stochastic frontier approach based on the Aigner and Chu’s [34] translation of Farrell’s frontier into a production function (SFA), which has been widely used in econometrics to estimate economic efficiency, technical efficiency, and allocative efficiency. Farrell defined economic efficiency as the product of both technical and allocative efficiency, where technical efficiency is the ratio of observed output to the corresponding limit of output depending on the input quantity used [35,36]. Allocative efficiency was defined as the ability of a farmer to use inputs to the level at which the value of the marginal product is equal to the cost of factors of production [35]. On the same note, profit efficiency combines both allocative and technical efficiency which describes the ability of the farm to attain the highest possible profit depending on the prices and level of inputs at that farm [37,38]. The SFA uses a parametric model to estimate the upper limit of the production frontier in agricultural production systems. The stochastic frontier production function has a regression type with a composite disturbance term. The likelihood function accommodates the variance parameters, with values between 0 and 1, indicating noise or profit inefficiencies. This model is recommended for farm-level data analysis when measurement errors, missing data, and risk variables significantly impact output [39–41].

The SFA always involves two approaches the One-step and Two-step approach in estimating the efficiency, the “Two-step” approach focuses on estimating the profit inefficiency and using it as the dependent variable in the second stage. This approach leads to skewed conclusions and diverging function distributions [42]. In response, Coelli and Battese [43] introduced the “One-step” approach, which involves estimating the stochastic frontier function and profit efficiency models simultaneously, allowing for a more precise prediction of profit inefficiency. The profit inefficiency in this context would be profit lost for failure to operate on the frontier [37,38]. This study adopted the One-step approach. The study analyzed the profitability of smallholder dairy production systems using gross margin (GM) analysis (equation (2)), a useful planning tool for small enterprises with limited fixed capital [44,45].


$$\begin{array}{c} {GM}_{jk}={TR}_{jk}-{TVC}_{jk} \end{array}$$ (2)

Where: GM is the gross margin. k represents the $k^{th}$ observation while j represents the $j^{th}$ dairy farmer. TR is total revenue and was calculated following equation (3). TVC is the total variable cost and was computed following equation (4).


$$\begin{array}{c} {TR}_{jk}=\sum PQ \end{array}$$ (3)

$$\begin{array}{c} {TVC}_{jk}=H_{Feedj}N_{Feedj}+H_{Vetj}N_{Vetj}+H_{Labourj}N_{Labourj}+\ldots +H_{n}N_{n} \end{array}$$ (4)

Where: Q is the quantity of milk sold in liters, and P is the price per liter of milk. N is the quantity of variable inputs, and H is the price of the variable inputs.

The Return on Investment (ROI) was calculated as suggested by Ojiakor et al. [46] to determine the level of profitability. As the ROI rises, profitability rises as well. ROI is depicted as follows in equation (5):


$$\begin{array}{c} ROI=\frac{GM}{TVC} \end{array}$$ (5)

Following the profitability analysis, the formulation of a stochastic frontier model follows from equation (6)


$$\begin{array}{c} Y=\beta_{\text{0}}X_{\text{1}}^{\beta_{\text{1}}}X_{\text{2}}^{\beta_{\text{2}}}X_{\text{3}}^{\beta_{\text{3}}}X_{\text{4}}^{\beta_{\text{4}}}X_{\text{5}}^{\beta_{\text{5}}}e^{ℰij} \end{array}$$ (6)

Where: Y represents the unit profit from milk production (UGX/Litre), $X_{\text{1}}$ represents the size of the land (a fixed input), $X_{\text{2}}$ represents the unit cost of feeds (UGX/Kg), $X_{\text{3}}$ represents the unit cost of veterinary services (UGX/Man-day), $X_{\text{4}}$ represents the unit cost of labor (UGX/Man-day), and $X_{\text{5}}$ represent cost of equipment for milking and drinking (UGX/Man-day).

As illustrated in equation (7), the profit function was linearized by applying natural logarithms to both sides.


$$\begin{array}{c} InY=\beta_{\text{0}}+\beta_{\text{1}}InX_{\text{1}}+\beta_{\text{2}}InX_{\text{2}}+\beta_{\text{3}}InX_{\text{3}}+\beta_{\text{4}}InX_{\text{4}}+\beta_{\text{5}}InX_{\text{5}}+\varepsilon_{ij} \end{array}$$ (7)

$$\begin{array}{c} \varepsilon_{ij}=v_{ij}-u_{ij} \end{array}$$

$\varepsilon_{ij}$= composite error term, $v_{ij}$= random error, and $u_{ij}$= dairy farm inefficiency

Using the inefficiency effects model stated in equation (8), the profit inefficiency is estimated


$$\begin{array}{c} u_{ij}=\delta_{\text{0}}\sum_{z=\text{1}}^{n}\delta_{z}{\delta X}_{zj} \end{array}$$ (8)

Where $\delta_{\text{0}}$ stands for the constant term, $\delta_{z}$ represents the parameters to be estimated, $u_{i}$ stands for profit inefficiency, and ${\delta X}_{zj}$ represents the vector of $z^{th}$ independent variables of the $j^{th}$ dairy farm. These variables include dairy farming experience (years), family size (number), education level (years), land size (acres), gender (male or female), access to credit, access to extension service, farmers’ group membership, age (years), breed type and quantity of water (Litres/cow). These variables and their *apriori* expectations are presented in Table 2.

**Table 2:** The list and descriptions of the variables used in profit efficiency analysis.

Variables	Measurement units	A *priori* expectation
**Household characteristics**		
Age	Years	+/-
Gender	Female = 0 and Male = 1	+
Farmers’ education level	Years	+/-
Farming experience	Years	+/-
Family size	Number of persons	+
Marital status	Single, married, divorced, widow, widower	+
Income level	UGX	+
**Production and market variables**		
Land size	Acres	+/-
Herd size	Number of herds	+/-
Lactating cows’ size	Number of lactating cows	+/-
Quantity of water provided	Quantity of water per cow in liters	+/-
Capital	UGX	+
Price of feeds	UGX/kg	+/-
Cost of veterinary services	UGX/man-day	+/-
Human labor	UGX/man-day	+
Cost of equipment	UGX/man-day	+/-
Quantity of output	Liters/cow	+
Quantity of output sold	Liters	+/-
Total cost of production	UGX	+/-
Total revenue for the product	UGX	+/-
Total profit	UGX	+
**Institutional variables**		
Market access information	Dummy (Yes = 1, No = 0)	+
Distance to the market	km	
Credit access	Dummy (Yes = 1, No = 0)	+
Group membership	Dummy (Yes = 1, No = 0)	+

To account for the two variance parameters, $\delta^{\text{2}}=\delta_{u}^{\text{2}}+\delta_{v}^{\text{2}}$ and $\gamma =\delta_{u}/\delta_{v}$ in the stochastic frontier production function, Aigner et al. [33] advise employing a likelihood function. Gamma (γ*)* values must fall between 0 and 1, with values of 0 indicating that all deviations from the frontier are solely caused by noise and values of 1 suggesting that all deviations are caused by profit inefficiencies.

If the Gamma (γ*)* value is close to 0, then the acceptable test statistic, according to Kumbhakar et al. [47], is the likelihood ratio (LR) test statistic [LR = −2($L_{R}-L_{U}$)], where, $L_{R}$, $L_{U}$ are the log-likelihood values calculated from the restricted (GLM) model and unrestricted stochastic model. Cobb-Douglas production is a restricted model measured by the general linear model (GLM) and the stochastic frontier model is the unrestricted model. The difference in their statistical value is contrasted with the crucial value for mixed distribution from the chi-square table at a particular degree of freedom reflecting the number of inefficient variables with a given level of significance [47].

To ensure robustness and reliability of the estimated frontier model, several diagnostic tests were conducted before estimation. These included tests for multicollinearity, heteroscedasticity, model specification errors, and distributional assumptions of the inefficiency term. In addition, likelihood ratio tests were employed to confirm the existence of inefficiency effects and to compare the suitability of the Cobb-Douglas and Translog specifications. This rigorous estimation strategy ensured that the selected model adequately captured the heterogeneous production conditions characterizing smallholder dairy farming systems.

### 2.5. Model specification test between Cobb-Douglas and translog profit frontier models

To identify the most appropriate functional form for estimating profit efficiency among smallholder dairy farmers, both the Cobb-Douglas and Translog stochastic frontier specifications were estimated and systematically compared using established econometric and statistical criteria. The evaluation emphasized model flexibility, goodness of fit, likelihood values, and information criteria. The Cobb-Douglas specification yielded a log-likelihood value of −271.792, whereas the Translog specification produced a higher log-likelihood of −264.355. Since higher log-likelihood values indicate a better fit to the observed data, the Translog model demonstrated stronger explanatory power than the Cobb-Douglas model.

A similar pattern emerged when considering the Akaike Information Criterion (AIC), which balances model fit against complexity. The Translog model achieved a lower AIC value (554.710) compared to the Cobb-Douglas model (557.584). This result reinforces the preference for the Translog specification in capturing overall dairy production efficiency, as the lower AIC suggests a more robust model [48].

However, when disaggregated by production system, the results reveal a more nuanced picture. For specific dairy production systems, the Cobb-Douglas specification consistently outperformed the Translog model (Table 3). These findings suggest that while the Translog specification is superior in aggregate, the Cobb-Douglas model provides a more appropriate fit within individual production systems, likely due to its simpler structure and reduced parameterization requirements.

**Table 3:** Disaggregated model diagnostic test.

Dairy Production System	Cobb-Douglas AIC	Translog AIC	Preferred Model
Zero-grazing	2.7605	2.7812	Cobb-Douglas
Tethered	3.0774	3.2210	Cobb-Douglas
Herding	2.7717	2.9130	Cobb-Douglas

To ensure robustness, several diagnostic tests were conducted. The Jarque-Bera normality test yielded a chi-square statistic of 0.5377 (p > 0.05), confirming that the data are normally distributed. The variance inflation factor (VIF) across all systems was consistently below 10, indicating the absence of multicollinearity. Finally, White’s test for heteroscedasticity produced a chi-square value of 0.3383 (p > 0.05), suggesting homoscedasticity across the three production systems.

Taken together, these results highlight the importance of tailoring functional form selection to the production context. While the Translog model offers greater flexibility and explanatory power at the aggregate level, the Cobb-Douglas specification remains more suitable for system-specific analyses of smallholder dairy production.

## 3. Results

### 3.1. Characteristics of smallholder dairy production systems

Table 4 describes the continuous socio-economic characteristics of dairy farmers by production system using one-way ANOVA. 39% used zero-grazing, 36% tethered, and 25% herding. Herding farmers were the oldest (average age 50) and most experienced (9 years). Zero-grazing farmers had higher education levels (8 years on average). Family sizes were 8 people across all systems. Herding farmers owned the most land (10 acres) while zero-grazing farmers owned the least (5 acres). Herding had larger herd sizes, while zero-grazing produced more milk per cow due to better care. Tethered farmers travelled the shortest distance to the market (2 km).

**Table 4:** Continuous socio-economic characteristics of smallholder dairy farmers in Gulu city and Gulu district using one-way ANOVA.

	Overall (N = 191)	Zero-grazing (n = 75)	Tethered (n = 69)	Herding (n = 47)	F-test	P-valve
Numerical Variables	Mean (SD)					
Age (years)	46.91(12.03)	47.59(11.46)	43.99(11.49)	50.13(12.92)	3.959**	0.021
Dairy Experience (years)	6.53(4.76)	5.67(4.24)	6.06(4.84)	8.60(4.94)	6.325***	0.002
Education level (years)	8.49(5.36)	10.03(5.74)	6.64(4.79)	8.77(4.77)	7.779***	0.001
Family size	7.82(2.91)	7.81(3.14)	7.51(2.41)	8.30(3.18)	1.032	0.358
Land size under dairy (acres)	1.43(3.42)	0.92(1.09)	1.11(2.38)	2.69(5.97)	4.494**	0.012
Total land size owned (acres)	6.93(12.00)	4.81(5.52)	7.14(14.05)	10.02(15.40)	2.788**	0.064
Herd size	5.56(4.58)	4.21(3.20)	3.87(2.20)	10.19(5.82)	47.766***	0.000
Number of lactating cows	1.95(1.46)	1.49(0.76)	1.59(0.75)	3.19(2.23)	29.294***	0.000
Qty of milk per cow per day (liter)	6.88(4.46)	10.40(3.72)	4.94(3.59)	4.13(2.71)	65.010***	0.000
Milking circle	7.10(1.44)	7.95(0.90)	6.17(1.50)	7.11(1.20)	38.166***	0.000
Calving interval	12.17(1.25)	12.19(1.39)	12.04(0.21)	12.32(1.80)	0.693	0.502
Qty of water per cow per day (liter)	25.70(8.45)	27.92(8.16)	25.51(8.96)	22.45(7.06)	6.445***	0.002
Distance to market (Km)	2.72(1.80)	3.17(1.78)	1.96(1.53)	3.11(1.86)	10.564***	0.000

The figures in parentheses represent mean & standard errors. *, ** and *** indicate level of significant at 10%, 5% and 1%.

Table 5 describes the categorical characteristics of the smallholder dairy farmers in the area using the chi-square test. The results showed that 64% of zero grazers were female and 81% were married, with gender being statistically significant (p < 0.01). Marital status did not vary across systems. Market access and credit access were crucial and statistically significant (p < 0.01 and p < 0.05, respectively). A significant number of zero grazers were under the group with a significant (p < 0.05). In tethered systems, 62% used hired labor, and 93% had second jobs, all at (p < 0.01).

**Table 5:** Categorical characteristics of dairy farmers compared by production system using the chi-square test.

Categorical Variables Category		Overall (N = 191)	Zero-grazing (n = 75)	Tethered (n = 69)	Herding (n = 47)	$x^{2}$	P-value
		Frequency (%)					
Gender	Male	88(46.1)	27(36.0)	29(42.0)	32(68.1)	12.832***	0.002
	Female	103(53.9)	48(64.0)	40(58.0)	15(31.9)		
Marital status	Married	153(80.1)	61(81.3)	55(79.7)	37(78.7)	134	0.935
	Otherwise	38(19.9)	14(18.7)	14(20.3)	10(21.3)		
Market access	Yes	131(68.6)	61(81.3)	39(56.5)	31(66.0)	10.782***	0.005
	No	60(31.4)	14(18.7)	30(43.5)	16(34.0)		
Credit access	Yes	116(60.7)	53(70.7)	35(50.7)	28(59.6)	6.084**	0.048
	No	75(39.3)	22(29.3)	34(49.3)	19(40.4)		
Extension services	Yes	185(96.9)	74(98.7)	66(95.7)	45(95.7)	1.492	0.474
	No	6(3.1)	1(1.3)	3(4.3)	2(4.3)		
Group membership	Yes	147(77.0)	64(85.3)	53(76.8)	30(63.8)	7.394**	0.025
	No	44(23.0)	11(14.7)	16(23.2)	17(36.2)		
Breed type	Friesian	95(49.7)	72(96.0)	17(24.6)	6(12.8)	126.637***	0.000
	Crossbreed	96(50.3)	3(4.0)	52(75.4)	41(87.2)		
Secondary occupation	Yes	171(89.5)	61(81.3)	64(92.8)	46(97.9)	10.333***	.006
	No	20(10.5)	14(18.7)	5(7.2)	1(2.1)		
Source of labor	Family	104(54.5)	44(58.7)	26(37.7)	34(72.3)	14.705***	0.001
	Hired	87(45.5)	31(41.3)	43(62.3)	13(27.7)		
Source of water	Tap	21(11.0)	18(24.0)	2(2.9)	1(2.1)	58.745***	0.000
	Borehole	79(41.4)	40(53.3)	33(47.8)	6(12.8)		
	Stream	91(47.6)	17(22.7)	34(49.3)	40(85.1)		
Form of milk sold	Raw	170(89.0)	72(96.0)	52(75.4)	46(97.9)	20.408***	0.000
	Processed	21(11.0)	3(4.0)	17(24.6)	1(2.1)		

The figures in parentheses represent frequency & percentages. *, ** and *** indicate level of significant at 10%, 5% and 1%.

### 3.2. Cost and return on dairy production

The study evaluated the costs and returns of three dairy production systems based on 2022 prices as shown in Table 6 using one-way ANOVA. Zero-grazing had the highest feed cost (983,800 UGX/US$265.89) and overall veterinary cost (222,202 UGX/US$60.05). The herding system incurred the highest equipment costs (71,255 UGX/US$19.26). Tethered grazers had the lowest labor costs, and the overall average labor cost was (608,330 UGX/US$164.41). Zero-grazing produced the most milk (14 liters), but milk prices were lower. The Zero-grazing system had the highest gross margin (3,374,447 UGX/US$912.01), while ROI was the lowest. Despite higher costs, dairy production was profitable with an average ROI of 2.07.

**Table 6:** Summarizes information on cost and return on dairy production using one-way ANOVA.

Numeric variables	Overall (N=191)	Zero-grazing (n=75)	Tethered (n=69)	Herding (n=47)	F-test	P-value
	Mean (SD)					
Feed cost	$130.65(196.86)	$265.89(234.39)	$34.86(78.85)	$55.50(114.67)	41.902***	0.000
Vet Cost	$60.05(47.85)	$84.77(48.64)	$34.27(35.21)	$58.47(42.68)	25.140***	0.000
Equipment cost	$15.66(18.51)	$16.53(20.74)	$12.26(10.40)	$19.26(23.06)	2.160	0.118
Labor cost	$164.41(100.15)	$200.49(113.26)	$13.68(61.12)	$181.31(94.82)	16.781***	0.000
Transport cost	$70.34(177.29)	$94.95(129.04)	$49.35(240.25)	$61.87(117.68)	1.263	0.285
Selling price	$0.46(0.12)	$0.43(0.08)	$0.48(0.15)	$0.46(0.12)	4.200**	0.016
Qty of milk sold (liters)	10.48(8.56)	13.99(8.57)	6.63(5.56)	10.54(9.84)	15.252***	0.000
Total cost	$370.79(27.56)	$567.68(284.64)	$195.08(140.83)	$314.55(203.04)	52.761***	0.000
Total Revenue	$1114.28(1035.39)	$1479.69(1056.46)	$670.17(661.78)	$1183.16(1211.06)	12.464***	0.000
Gross Margin	$743.49(861.71)	$912.01(913.65)	$475.1(560.32)	$868.61(1043.89)	5.528***	0.005
Return on investment	2.07(1.81)	1.66(1.34)	2.31(2.12)	2.36(1.89)	3.168**	0.044

The figures in parentheses represent mean & standard errors. *, ** and *** indicate level of significant at 10%, 5% and 1% $1 = 3700UGX by the time of data collection.

### 3.3. Stochastic profit frontier for dairy production systems

Table 7 presents the estimates of the stochastic profit frontier model and inefficiency effects for the overall sample and across the three dairy production systems, namely zero-grazing, tethering, and herding systems. The results reveal substantial differences in the determinants of profit efficiency across the dairy production categories, highlighting the heterogeneous nature of smallholder dairy farming systems.

**Table 7:** Results of the Stochastic Profit frontier for dairy production systems.

	Overall (N = 191)		Zero-grazing (n = 75)		Tethered (n = 69)		Herding (n = 47)	
Variables	Coefficients	Standard error	Coefficients	Standard error	Coefficients	Standard error	Coefficients	Standard error
**Profit efficiency**								
Constant	2.996**	1.398	8.865***	2.941	−1.058	3.024	−7.284**	4.204
Land size	0.075	0.082	0.022	0.104	0.343**	0.181	−0.482***	0.130
Cost of feeds	−0.145***	0.052	−0.553***	0.141	0.235***	0.083	−0.132**	0.066
Vet cost	−0.049	0.083	−0.059	0.134	0.140	0.136	0.039	0.135
Cost of equipment	0.069**	0.041	−0.069	0.074	0.100	0.072	0.313**	0.178
Cost of labor	−0.015	0.090	−0.015	0.142	−0.344	0.246	0.480	0.300
Herd size	0.136	0.104	0.700***	0.203	0.146	0.238	0.175	0.252
**Inefficiency model**								
Constant	−0.739	1.699	4.993	5.526	−6.435**	2.687	−3.944	4.968
Age of dairy farmer	0.026	0.018	0.071**	0.042	0.013	0.031	−0.055***	0.017
Education level	−0.089**	0.046	−0.211**	0.120			−0.154***	0.040
Years of dairy experience			−0.152	0.132	−0.076	0.063	0.250***	0.072
Gender			1.586	1.111				
Family size	−0.099	0.071	−0.281**	0.167	0.649***	0.197	−0.801***	0.209
Marital status	0.985*	0.577	2.445*	1.376	1.019	0.812		
Distance to market (Km)	−0.361**	0.164	−0.762**	0.397	0.010	0.224	0.105	0.188
Off-farm income			−0.057	1.019	2.207	1.481	2.102	5.163
Extension services	0.082	0.064	−26.054	55.078	−2.081***	0.947		
Credit access	1.281**	0.647	19.079	54.222	1.296	1.036	−0.729	1.225
Group membership	−0.334	0.680	3.687	5.229			3.442**	1.550
Market access information	2.138**	1.002						
Breed type	−2.490**	1.110	−1.932	1.960			−1.350**	0.805
Quantity of water(liter)	0.277	0.311	0.009	0.037	−0.067***	0.030	3.649***	0.770
**Variances parameters**								
Usigma constant	0.666**	0.329	−0.616	0.862	−3.657	2.067	−2.283***	0.605
Vsigma constant	−1.993***	0.376	−1.162***	0.222	−0.268	0.171	−1.266***	0.286
Sigma-u	1.395***	0.230	0.735***	0.317	0.161	0.166	0.319***	0.097
Sigma-v	0.369***	0.069	0.559***	0.062	0.875***	0.075	0.531***	0.076
Lambda	3.779***	0.233	1.314***	0.344	0.184	0.178	0.601***	0.073
Log-likelihood	−234.0169		−74.1249		−89.1763		−36.1985	

The figures in parentheses represent coefficients & standard errors. *, ** and *** indicate level of significant at 10%, 5% and 1%

For all dairy production systems, equipment costs positively influenced profit efficiency (β = 0.313, p < 0.05), indicating that investment in production assets improves operational efficiency across the dairy production systems. Conversely, feed costs had a strong negative effect on profitability (β = −0.553, p < 0.01), implying that feed expenditure remains a major constraint to profit maximization across the dairy production systems.

Under the zero-grazing production system, herd size emerged as the most important positive determinant of profit efficiency (β = 0.700, p < 0.01). This result indicates that farmers operating larger dairy herds within confined production systems benefit from increased milk production and improved resource utilization. Conversely, feed costs had a strong negative effect on profitability (β = −0.553, p < 0.01), implying that feed expenditure remains a major constraint to profit maximization under zero-grazing dairy systems.

Within the tethering production system, land size positively and significantly influenced profit efficiency (β = 0.343, p < 0.05). This suggests that access to larger land holdings improves grazing opportunities and feed availability, thereby enhancing profitability. Feed costs also exhibited a positive and significant relationship with profit efficiency (β = 0.235, p < 0.01), indicating that farmers who invested more in feed supplementation were able to achieve better production outcomes and higher returns.

Under the herding production system, land size negatively and significantly affected profit efficiency (β = −0.482, p < 0.01), suggesting that larger grazing areas may be associated with extensive and less efficient production practices. Feed costs similarly reduced profitability (β = −0.132, p < 0.05), reflecting the increasing cost burden associated with feed acquisition. However, equipment costs positively influenced profit efficiency (β = 0.313, p < 0.05), indicating that investment in production assets improves operational efficiency even within extensive grazing systems.

### 3.4. Determinants of profit efficiency of smallholder dairy production systems

The inefficiency model explains the socioeconomic and institutional factors influencing deviations from the maximum attainable profit frontier. Negative coefficients imply reduced inefficiency (higher efficiency), whereas positive coefficients indicate increased inefficiency, as shown in Table 7. For all dairy production systems, education level significantly reduced profit inefficiency (β = −0.089, p < 0.05), suggesting that educated farmers are more capable of adopting improved management practices and making efficient production decisions. Distance to market also negatively affected inefficiency (β = −0.361, p < 0.05), implying that improved market proximity enhances farmers’ access to inputs, output markets, and market information.

Breed type negatively and significantly influenced inefficiency (β = −2.490, p < 0.05), indicating that improved dairy breeds contribute substantially to higher profit efficiency. In contrast, credit access and market information access positively influenced inefficiency, suggesting that although farmers accessed financial and market services, these resources may not have been effectively utilized toward productive dairy investments.

Within the zero-grazing system, age of the dairy farmer positively influenced inefficiency (β = 0.071, p < 0.05), indicating that older farmers were relatively less efficient than younger farmers, possibly due to slower adoption of improved dairy management practices. Education level and family size significantly reduced inefficiency, implying that educated households with adequate family labor achieved higher levels of efficiency. Distance to market positively affected efficiency by reducing transactions and transportation costs.

Under the tethering system, family size significantly increased inefficiency (β = 0.649, p < 0.01), suggesting that larger households may exert pressure on farm resources and reduce managerial efficiency. Extension services significantly reduced inefficiency (β = −2.081, p < 0.01), demonstrating the important role of agricultural advisory services in improving dairy farm management and productivity. Water availability negatively affected inefficiency (β = −0.067, p < 0.01), implying that adequate access to water improves animal productivity and farm performance.

For the herding production system, age of the farmer negatively influenced inefficiency (β=−0.055, p < 0.01), indicating that older farmers operating under extensive grazing systems benefited from accumulated indigenous knowledge and production experience. Education level and family size also significantly reduced inefficiency, highlighting the importance of human capital and household labor in traditional grazing systems. However, years of dairy farming experience were found to negatively influence profit efficiency (β = 0.250, p < 0.01), suggesting that farmers with longer experience in traditional herding practices were relatively less profit efficient compared to their counterparts. Similarly, group membership negatively affected profit efficiency under the herding system (β = 3.442, p < 0.05). Furthermore, the quantity of water negatively and significantly influenced profit efficiency under the herding production system (β = 3.649, p < 0.01), indicating that increased water use was associated with lower levels of profit efficiency among herding farmers. Although water is an essential input in dairy production, the negative relationship may reflect the high costs and inefficiencies associated with water access in herding grazing systems.

### 3.5. Hypothesis testing for the present profit inefficiency

A likelihood ratio (LR) test was used in Table 8 to examine the null hypothesis for profit efficiency (γ=0). The variance of the parameter gamma (γ), which was also determined to be 0.93454 with statistical significance (p < 0.001), indicated that 93.45% of the technical, allocative, scale inefficiency, and the remaining 6.55% of the inefficiency was due to random error brought about by weather changes and other external factors not considered by the model. This implies that profit inefficiency accounts for 93.45% of the dairy production output across the three dairy production systems. Therefore, the null hypothesis (γ=0), does not count.

**Table 8:** The null hypothesis for profit efficiency.

Production systems	Variable	Parameter	Coefficient	P > ׀z׀
Overall dairy production system	$\sigma^{\text{2}}$ **=** $\sigma_{u}^{\text{2}}$ **+** $\sigma_{v}^{\text{2}}$	$\sigma^{\text{2}}$	2.08281	0.000
	γ**=**$\sigma_{u}^{\text{2}}$**/(**$\sigma_{u}^{\text{2}}$**+**$\sigma_{v}^{\text{2}}$)	γ	0.93454	0.000
	**Log-likelihood**			
	$L_{R}$		−271.792	0.000
	$L_{U}$		−234.069	0.000
	**LR = −2(**$L_{R}-L_{U}$)	LR	75.5498	0.000
Zero-grazing system	$\sigma^{\text{2}}$ **=** $\sigma_{u}^{\text{2}}$ **+** $\sigma_{v}^{\text{2}}$	$\sigma^{\text{2}}$	0.85303	0.000
	γ**=**$\sigma_{u}^{\text{2}}$**/(**$\sigma_{u}^{\text{2}}$**+**$\sigma_{v}^{\text{2}}$)	γ	0.63337	0.000
	**Log-likelihood**			
	$L_{R}$		−96.5189	0.000
	$L_{U}$		−74.1249	0.000
	**LR = −2(**$L_{R}-L_{U}$)	LR	44.7880	0.000
Tethered system	$\sigma^{\text{2}}$ **=** $\sigma_{u}^{\text{2}}$ **+** $\sigma_{v}^{\text{2}}$	$\sigma^{\text{2}}$	0.79099	0.000
	γ**=**$\sigma_{u}^{\text{2}}$**/(**$\sigma_{u}^{\text{2}}$**+**$\sigma_{v}^{\text{2}}$)	γ	0.03263	0.000
	**Log-likelihood**			
	$L_{R}$		−99.1720	0.000
	$L_{U}$		−89.1763	0.000
	**LR = −2(**$L_{R}-L_{U}$)	LR	19.9914	0.000
Herding system	$\sigma^{\text{2}}$ **=** $\sigma_{u}^{\text{2}}$ **+** $\sigma_{v}^{\text{2}}$	$\sigma^{\text{2}}$	0.38402	0.000
	γ**=**$\sigma_{u}^{\text{2}}$**/(**$\sigma_{u}^{\text{2}}$**+**$\sigma_{v}^{\text{2}}$)	γ	0.26542	0.000
	**Log-likelihood**			
	$L_{R}$		−58.1355	0.000
	$L_{U}$		−36.1985	0.000
	**LR = −2(**$L_{R}-L_{U}$)	LR	43.8740	0.000

The figures in parentheses represent coefficients & p-values.

### 3.6. Profitability of the sampled dairy farmers

The findings in Table 9 showed how profitable the sampled smallholder dairy producers were under various dairy production techniques. With a mean efficiency score of 0.6121, the overall profit efficiency ranges from 0.0552 to 0.8287. The typical smallholder dairy farmer in the sample could only improve production by 26.14 percent (0.8287–0.6121)/0.8287) to reach the profit efficiency level of the most efficient farmers. The least effective smallholder farmer can improve production by 93.34% (0.8287–0.0552)/0.8287) to match the most effective smallholder dairy farmer’s needed profit efficiency. Only 1.57 percent of dairy producers who are smallholders operate at an efficiency level of 0.81 to 0.90. The smallholder farmers under the zero-grazing system had the highest mean profit efficiency score of 0.9886 compared to the tethered and herding system, with the range of efficiency scores from 0.9881 to 0.9890.

**Table 9:** Profitability of the sampled smallholder dairy producers under various dairy production systems.

	Overall (N = 191)	Zero-grazing (n = 75)	Tethered (n = 69)	Herding (n = 47)
**Efficiency score**	**Frequency (%)**			
<0.40	19(9.95)	0(0.00)	45(65.22)	24(51.06)
0.41-0.50	19(9.95)	0(0.00)	7(10.14)	3(6.38)
0.51-0.60	36(18.85)	0(0.00)	4(5.80)	1(2.13)
0.61-0.70	59(30.89)	0(0.00)	4(5.80)	2(4.26)
0.71-0.80	55(28.80)	0(0.00)	1(0.14)	3(6.38)
0.81-0.90	3(1.57)	0(0.00)	0(0.00)	5(10.64)
>0.90	0(0)	75(100.00)	8(11.59)	9(19.15)
Mean	0.6121	0.9886	0.3572	0.5163
Std. Dev	0.1492	0.0002	0.2869	0.3260
Minimum	0.0552	0.9881	0.0208	0.0050
Maximum	0.8287	0.9890	0.9999	0.9833

The figures in parentheses represent frequency & standard percentages.

## 4. Discussions

### 4.1. Smallholder dairy farmers’ characteristics under different production systems

The study found that smallholder dairy farmers in northern Uganda were predominantly middle-aged, suggesting that they were capable of adopting modern farming practices. Our results were corroborated by Mugumaarhahama et al.’s [49] findings which also observed a dominance of middle-aged farmers in the dairy industry. However, the findings contradicted those of Maina et al. [50], who reported an older population dominating the dairy industry. Herding farmers, in particular, had more years of experience, which contributed to a better understanding of local conditions and effective livestock management, leading to improved productivity and resilience. These findings align with Benon et al. [51], who noted that farmers in Gulu were still new to certain dairy production systems, like zero-grazing.

The study also revealed that zero-grazing farmers in northern Uganda were generally more educated than their herding counterparts. This education is necessary for modern dairy farming practices, such as animal nutrition and health management. The findings were consistent with the findings of García et al. [52], who reported that low education levels correlate with low milk yields. However, the findings contradicted Hansen et al. [53], who observed that higher education could reduce participation in dairy production. Furthermore, herding farmers were found to have larger land sizes than zero-grazing farmers, as herding systems require more land for grazing while zero-grazing systems confine cattle to smaller spaces. Land is considered a communal resource in the region [54], which is more conducive to herding practices.

In terms of herd management, herding farmers had larger herds and more lactating cows compared to zero-grazing farmers. This was due to the extensive nature of herding systems, which utilize larger land areas and indigenous cattle breeds that adapt better to local conditions, leading to higher survival rates. However, the milk yield per cow was relatively low [55]. The study also found that herding was a male-dominated activity, consistent with traditional gender roles in the region, where men typically manage livestock [56]. This contrasted with the findings of Korir et al. [57], who reported a greater female participation in dairy production. Zero-grazing farmers had better access to markets and credit, with cooperatives facilitating their participation in the milk trade, aligning with [58].

Finally, the study found that zero-grazing farmers in northern Uganda were more likely to be in groups, helping them sell their milk, access resources, and learn from each other. Herding farmers, on the other hand, were less likely to participate in such groups due to the more traditional nature of their farming systems. This finding agreed with those of Grace et al. [59], who studied group membership and food safety in Nigeria. Zero-grazing farmers were also more likely to have Friesian cows, which are better suited for milk production and can be managed more efficiently in zero-grazing systems, this finding coincided with Benon et al. [51], who observed that exotic breeds dominate milk production. Additionally, zero-grazing farmers used less family labor, focusing on efficiency, whereas herding farmers employed more family labor due to the labor-intensive nature of their practices. These findings supported [60], who noted that family labor was often insufficient for running dairy production units.

### 4.2. Stochastic profit frontier across dairy production systems

The stochastic profit frontier results revealed substantial variation in the determinants of profit efficiency across dairy production systems, confirming the heterogeneous nature of smallholder dairy farming. The findings imply that factors influencing profit efficiency differ according to the management intensity, resource endowment, and production practices characterizing each dairy production system.

For the overall dairy production systems, equipment costs positively and significantly influenced profit efficiency. This finding suggests that investment in productive dairy assets such as milking equipment, feeding facilities, water infrastructure, and housing structures enhances operational efficiency and productivity among dairy farmers. The result supports the argument that capital investment improves production management and reduces inefficiencies associated with traditional production methods. Similar findings were reported by Tafesse et al. [61] in cassava production studies in Ethiopia, where investment in farm technologies significantly improved production efficiency. Likewise, Holohan et al. [18] observed that access to productive assets improves resource allocation and enhances farm profitability among smallholder farmers.

Conversely, feed costs negatively affected profit efficiency under overall dairy production system, indicating that rising feed expenditures substantially reduced dairy profitability. Feed is one of the highest recurrent costs in dairy production, particularly under smallholder systems where commercial feed prices are increasingly volatile. This finding is consistent with studies by Sarica et al. [62] and Ragkos et al. [63], who both independently reported that high feeding costs significantly reduce technical and economic efficiency among dairy farmers. Similarly, Holohan et al. [18] found that feed-related expenditures constitute a major constraint to profitability in livestock production systems.

#### 4.2.1. Stochastic profit frontier for the zero-grazing system.

Under the zero-grazing production system, herd size positively influenced profit efficiency, suggesting that farmers with larger herds benefit from economies of scale, improved milk output, and better utilization of fixed production resources. In intensive dairy systems, herd expansion often enhances specialization and allows farmers to distribute operational costs across greater production volumes. This finding agrees with the works of Næss & Ba˚rdsen [64], and Jago & Berry [65], who both independently reported that herd size positively contributes to farm efficiency through increased productivity and improved resource use.

However, feed costs negatively affected profit efficiency under the zero-grazing system. Zero-grazing systems are highly dependent on purchased feeds and supplementary fodder, making feed expenditure a major operational burden. The result supports findings by Holohan et al. [18] and Datta et al. [44], who observed that intensive livestock systems experience declining profitability when feed costs increase disproportionately relative to milk output.

#### 4.2.2. Stochastic profit frontier for the tethering system.

Within the tethering production system, land size positively influenced profit efficiency. This suggests that access to larger landholdings improves feed availability through natural grazing and fodder cultivation, thereby reducing dependence on purchased feed inputs. The result is consistent with earlier findings [66–68], that noted that land availability positively affects agricultural efficiency by enhancing access to productive resources. This contrasts with the findings of Maina et al. [50] who asserted that land size reduces the efficiency of smallholder farmers.

Feed costs also positively influenced profit efficiency under the tethering system, implying that farmers who invested more in supplementary feeding achieved higher milk yields and better economic returns. Unlike the zero-grazing system where feed costs reflect production constraints, feed expenditure under tethering may represent productive investment in animal nutrition. This finding supports the argument by Ragkos et al. [63] that strategic investment in productivity-enhancing inputs can improve farm efficiency and profitability.

#### 4.2.3. Stochastic profit frontier for the herding system.

Under the herding production system, land size negatively affected profit efficiency, suggesting that larger grazing areas may encourage extensive and less intensive production practices. Farmers operating across large grazing areas may face difficulties monitoring livestock, controlling diseases, and efficiently managing feed resources. Similar findings were reported by Maina et al. [50], who observed that larger landholdings under traditional production systems may reduce managerial efficiency due to extensive resource utilization, and this contradicts the findings of Girma & Ayalew [66], Kumar & Moharaj [67] and Gaspard et al. [68] who argued that land size positively influence the efficiency of smallholder livestock farmers.

Feed costs also negatively affected profit efficiency under the herding system, reflecting the increasing cost burden associated with feed acquisition during dry seasons and periods of pasture scarcity. This finding aligns with studies by Federico [69], who found that feed shortages and high supplementary feeding costs reduce livestock profitability in extensive grazing systems.

Nevertheless, equipment costs positively influenced profit efficiency under the herding system. This implies that investment in productive assets such as water storage facilities, transportation equipment, and livestock handling infrastructure improves operational performance even in extensive grazing systems. The result confirms the importance of technological and infrastructural investment in improving efficiency across diverse livestock production systems [61].

### 4.3. Determinants of profit efficiency of smallholder dairy production systems

The inefficiency model identified several socioeconomic and institutional factors influencing deviations from the maximum attainable profit frontier. For the all dairy production systems, education levels significantly reduce profit inefficiency, indicating that educated farmers are more capable of adopting improved technologies, utilizing production information, and making efficient farm management decisions. This finding is consistent with studies by Ruzhani & Mushunje [70], and Manyike et al. [71]**,** who argued that education improves managerial ability and resource allocation efficiency in agricultural production systems. Similar findings were also reported by Bayiyana et al. [72], who found that education enhances production efficiency through improved decision-making and technology adoption, but contradict the findings of Maina et al. [73] who found education to negatively impact efficiency.

Distance to market negatively affected inefficiency, implying that improved market proximity enhances access to inputs, extension services, and output markets. This finding agrees with van der Lee et al. [74], and Migose et al. [45], who observed that market accessibility reduces transaction costs and improves production efficiency among smallholder farmers.

Breed type also reduced inefficiency, indicating that improved dairy breeds contribute significantly to higher productivity and profitability. Improved breeds generally produce higher milk yields and respond better to modern feeding and veterinary practices. Similar findings were documented by Chawala et al. [75], and Acharya et al. [76], who found that improved livestock technologies positively influence farm efficiency.

However, credit access and market information positively influenced inefficiency in the overall dairy production system. This finding contradicts several earlier studies which reported positive efficiency effects of financial and market services. For example, Haryanto et al. [77], and Zhang et al. [78] found that access to credit improves farmers’ ability to purchase productive inputs and adopt improved technologies. The contrasting result in this study may indicate ineffective utilization of borrowed funds, diversion of credit toward non-farm activities, or limited relevance of available market information to dairy production decisions.

#### 4.3.1. Determinants of profit efficiency in the zero-grazing system.

Within the zero-grazing system, older farmers were relatively less efficient than younger farmers. Younger farmers may be more willing to adopt improved dairy technologies and commercial production strategies compared to older farmers who tend to rely on conventional management practices. This finding supports the works of Bayiyana et al. [72], and Stein & Amanda [79], who observed that younger farmers are generally more innovative and responsive to technological change.

Education level and family size significantly reduced inefficiency under the zero-grazing system. Educated households with sufficient family labor are more capable of managing labor-intensive dairy activities such as feeding, milking, and hygiene management. Similar findings were reported by Bayiyana et al. [72], who found that household labor availability improves efficiency among smallholder farmers. This contradicts the findings of Maina et al. [73] who reported that education has a negative impact on the efficiency of smallholder farmers.

#### 4.3.2. Determinants of profit efficiency in the tethering system.

Under the tethering system, family size positively influenced inefficiency, suggesting that larger households may exert pressure on limited farm resources and reduce managerial efficiency. This finding contrasts with studies that view family labor as productive agricultural capital but agrees with research by Bitana et al. [80], who found that large household dependency burdens may reduce production efficiency.

Extension services significantly reduced inefficiency under the tethering system, demonstrating the importance of agricultural advisory services in improving dairy management practices, disease control, and feed utilization. This finding supports studies by Mungai et al. [81], who found that extension access improves farm efficiency through knowledge transfer and technical support.

Water availability also reduced inefficiency under the tethering system, highlighting the importance of reliable water access in livestock productivity. Adequate water improves animal health, milk production, and feed utilization efficiency [82,83].

#### 4.3.3. Determinants of profit efficiency in the herding system.

For the herding system, older farmers were more efficient than younger farmers, suggesting that accumulated indigenous knowledge and long-term livestock management experience enhance efficiency under traditional grazing systems, collaborating with the findings of Borda [84]. This finding differs from the zero-grazing system, emphasizing the contextual nature of production efficiency determinants across dairy systems.

Education levels were positively linked with profit efficiency, educated farmers are better equipped to manage farms effectively and adopt modern technologies, these findings aligned with Bayiyana et al. [72], but contradict the findings of Maina et al. [73] who found that education negatively impacts efficiency.

Family size positively influenced profit efficiency under the herding production system, indicating that larger households provided essential family labor for herding, livestock management, and grazing supervision, thereby reducing labor costs and improving operational efficiency. This suggests that household labor remains a critical production resource in labor-intensive and low-mechanized dairy systems. The finding is consistent with Adewale et al. [85], who reported that larger household sizes enhance agricultural efficiency and profitability through improved labor availability.

However, years of dairy farming experience negatively influenced profit efficiency under the herding system. This suggests that farmers with prolonged exposure to traditional herding practices may be less likely to adopt improved production technologies and commercial dairy management practices. Previous studies have shown that reliance conventional livestock practices may reduce efficiency under changing production environments [85,86].

Group membership also negatively affected profit efficiency under the herding system. Although farmer groups are generally expected to improve access to information and markets, the result suggests that existing farmer organizations may not have effectively supported productive dairy investments or commercialization activities. Weak institutional coordination and limited functional effectiveness of farmer groups may explain this outcome [87]. This contradicts the findings of Manyike et al. [71] who asserted that a farmer’s group membership positively affects livestock production efficiency.

Finally, the quantity of water negatively influenced profit efficiency under the herding system. While water is essential for dairy production, the result may reflect the high costs and inefficiencies associated with water access in extensive grazing systems. Farmers operating under herding systems often travel long distances to access water sources, increasing labor requirements and operational costs. Consequently, increased water use may not necessarily translate into proportional productivity gains under traditional grazing systems [88].

## 5. Conclusions

A comparative analysis of smallholder dairy production systems in northern Uganda reveals significant differences in socio-economic and management factors among zero-grazing, tethered, and herding systems. Younger, educated farmers using zero-grazing systems achieve higher milk yields and profit efficiency, while traditional herding practices show inefficiencies. A stochastic profit frontier analysis identifies land size, feed and veterinary costs, and socio-economic factors like age, education, and extension services as key determinants of profitability. Tailored interventions, including improved market access, education, and effective agricultural extension programs, are critical to enhancing profit efficiency and reducing inefficiencies in smallholder dairy systems.

## 6. Recommendations and policy implications

The study recommends enhancing profit efficiency among smallholder dairy farmers in northern Uganda through targeted strategies. Expanding extension services, especially for zero-grazing and tethered systems, and offering training on modern dairy management, feed, and veterinary care are essential. Supporting younger farmers to adopt innovative practices through education and workshops is critical. Tailored financial products with flexible terms and financial literacy training can improve access to resources, while improved infrastructure and transportation will enhance market access. Promoting high-yielding breeds in zero-grazing and tethered systems is key to boosting productivity and ensuring sustainable and profitable dairy farming practices.
