# Supplementary material for: Profit efficiency of smallholder dairy production systems: Empirical evidence from Northern Uganda
Source: PLoS One. 2026 Aug 24;21(8):e0333337. doi: 10.1371/journal.pone.0333337 (PMC13502703; doi:10.1371/journal.pone.0333337)
Supplement: Supplementary file 1 [file pone.0333337.s001.docx]

**Comments to Authors**

*The manuscript presents an important comparative analysis of profit efficiency across three dairy production systems. However, there are several concerns regarding the overall strength of the manuscript.*

1. Can the Authors provide a map that clearly represents the study area?
2. Authors should recast lines 216 and 217. They may wish to adopt something like “Table 2 describes the…”
3. I can see that authors have more or less carried out a comparative study across three dairy practitioners: zero-grazing, Tethered, and herding. However, this very important consideration was not included in the abstract. Authors are therefore advised to consider rewriting the abstract to capture this. In addition, there should be a specific mention of results, especially figures/metrics.
4. Where is the formula adopted for the sample size selection?
5. Lines 117,118,119 – authors should recast to correct ambiguity. Could be restructured like this: A face-to-face interview was done using a structured questionnaire.
6. A lot of work has been done on all the types of efficiencies mentioned in the manuscript. Allocative, technical, and economic efficiencies. Also, SFA. Authors should cite and support old references with recent ones.
7. Authors should recast lines 228 and 229
8. Authors should endeavour to arrange their references in ascending order by year and not by alphabets
9. As much as possible, Authors should add the DOIs to references
10. In Table 4, the Equipment cost is not significant, Authors should endeavour to remove the asterisk
11. In section 2.3, the authors should update references with more recent ones; the references are too old. Other sections should be reviewed as well.
12. Line 136- Authors may wish to swap “serious statistical analysis” with “rigorous statistical analysis.”
13. Where are the diagnostic test results of the Chi-Square and the ANOVA? Authors wrote- “… and a one-ANOVA test.” Do you mean a one-way ANOVA? If your Chi-Square result is Table 3, please label it as such and describe the same in your write-up as well.
14. Lines 100-101 - If the Acholi region, where the study locations (Gulu City and Gulu District) are situated, has a low cattle population, what then is the underlying rationale for selecting this area for the study?
15. How were the authors able to just target 380 farmers? How and where did you obtain your population sample size. This should be described. Also, present the sample size table as written in Line 110
16. “divisions and sub-counties were stratified”. How many divisions and sub-counties were selected?
