# Supplementary material for: Profit efficiency of smallholder dairy production systems: Empirical evidence from Northern Uganda
Source: PLoS One. 2026 Aug 24;21(8):e0333337. doi: 10.1371/journal.pone.0333337 (PMC13502703; doi:10.1371/journal.pone.0333337)
Supplement: Supplementary file 2 [file pone.0333337.s002.docx]

**Point-by-point response to reviewers and editors**

**Date:** 31/May/2026

**Ref: Submission ID:** PONE-D-25-49874

**Title of Article:** Profit Efficiency of Smallholder Dairy Production Systems: Empirical evidence from northern Uganda

Dear Editor,

Dear Reviewers,

We are pleased to respond to the editorial and review comments raised. The revised manuscript submitted together with this response letter, has all changes highlighted in yellow.

Below is a detailed explanation of how each of the issues raise have been addressed.

| **S/N** | **Issue** | **Response** |
| --- | --- | --- |
|  | **Reviewer #1:** |  |
| 1 | Cobb-Douglas was used, but no justification or testing provided. It has some restrictive assumptions and may not hold across heterogeneous smallholder systems. A comparative test should be conducted. | Thank you for pointing this out. We have revised and included the justification for the use of Cobb-Douglas and comparative test was included.  To identify the most appropriate functional form for estimating profit efficiency among smallholder dairy farmers, both the Cobb-Douglas and Translog stochastic frontier specifications were estimated and systematically compared using established econometric and statistical criteria. The results are presented in However, when disaggregated by production system, the results reveal a more nuanced picture. For specific dairy production systems, the Cobb-Douglas specification consistently outperformed the Translog model.  Thus, it’s the results of the Cobb-Douglas model that are presented  See L295 – 214 and Table 3. |
| 2 | Gamma refers to what the gap between a farmer and the frontier is due to genuine inefficiency versus random noise (weather, measurement error, luck). Their gamma is 0.038 — meaning only 4% of that gap is inefficiency and 96% is just noise. Thus, there's almost no systematic inefficiency which means farmers are mostly affected by random factors. But authors are analysing what causes inefficiency — age, education, credit access, etc. Either acknowledge inefficiency or explain why the analysis is still valid despite the low gamma. | Thank you for this observation.  We have revised our analysis and corrected this error.  See L441-448 and Table 8. |
| 3 | Zero-grazing is presented in the paper as the better system due to better management, higher gross margins, etc., but in Table 7 it shows that zero-grazing farmers have the worst average efficiency score, while herding and tethered farmers score higher. This is not what the discussion claims. | We thank the reviewer for this important observation. We have revised and corrected the efficiency scores  See Table 9 |
| 4 | For each production system, they estimate a separate model with around 15 parameters. The herding group has only 47 farmers. You need at least 10 observations per parameter for stable estimates. With 47 farmers and 15 parameters, the estimates become unreliable. | We thank the reviewer for this important observation. We have revised and removed other variables  See Table 7 |
| 5 | The authors list many (20+) variables and test them, but no reasoning is provided to motivate why these variables matter for profit efficiency. Apply a conceptual framework to provide a motivation for including certain variables. | We thank the reviewer for this important observation. We have revised and included the conceptual framework to provide the motivation for including certain variables  See Figure 1 |
|  | **Reviewer #2:** |  |
| 1 | Can the Authors provide a map that clearly represents the study area? | Thank you for the feedback, we have revised and included and included the map of the study area |
| 2 | Authors should recast lines 216 and 217. They may wish to adopt something like “Table 2 describes the…” | We thank the reviewer for this valuable suggestion; we have revised and recasted lines 216 and 217  See L328 and L329 |
| 3 | I can see that authors have more or less carried out a comparative study across three dairy practitioners: zero-grazing, Tethered, and herding. However, this very important consideration was not included in the abstract. Authors are therefore advised to consider rewriting the abstract to capture this. In addition, there should be a specific mention of results, especially figures/metrics. | Thank you for the feedback, we have revised and included and included the results especially figures in the abstract  See Abstract |
| 4 | Where is the formula adopted for the sample size selection? | Thank for this observation. The sample size for this followed the Krejcie & Morgan (1970) table of sample size determination. This is presented in L128 - 138 |
| 5 | Lines 117,118,119 – authors should recast to correct ambiguity. Could be restructured like this: A face-to-face interview was done using a structured questionnaire. | We thank the reviewer for this valuable suggestion; we have revised and recasted lines 117, 118, and 119  See L179, L180, and L181 |
| 6 | A lot of work has been done on all the types of efficiencies mentioned in the manuscript. Allocative, technical, and economic efficiencies. Also, SFA. Authors should cite and support old references with recent ones. | We thank the reviewer for this important observation. We have cited and supported the old references with recent ones |
| 7 | Authors should recast lines 228 and 229 | We thank the reviewer for this valuable suggestion; we have revised and recasted lines 228 and 229  See L339 and L340 |
| 8 | Authors should endeavour to arrange their references in ascending order by year and not by alphabets | We thank the reviewer for this important observation. We have arranged the references in ascending order by year and not by alphabets |
| 9 | As much as possible, Authors should add the DOIs to references | We thank the reviewer for this important observation. We have added the DOIs to the references |
| 10 | In Table 4, the Equipment cost is not significant, Authors should endeavour to remove the asterisk | We have removed the asterisk  See Table 6 |
| 11 | In section 2.3, the authors should update references with more recent ones; the references are too old. Other sections should be reviewed as well. | We thank the reviewer for this observation and for highlighting the need for inclusion of recent references. We have revised and included the recent references |
| 12 | Line 136- Authors may wish to swap “serious statistical analysis” with “rigorous statistical analysis.” | We thank the review for this suggestion. We have revised and swapped “serious” with rigorous  See L198 |
| 13 | Where are the diagnostic test results of the Chi-Square and the ANOVA? Authors wrote- “… and a one-ANOVA test.” Do you mean a one-way ANOVA? If your Chi-Square result is Table 3, please label it as such and describe the same in your write-up as well. | We thank the reviewer for this important observation. We have revised and one-way ANOVA and Chi-square result in the table and describe the same in the write-up  See L328, L329, L335, L336, L339, L340, L346, and L347 |
| 14 | Lines 100-101 - If the Acholi region, where the study locations (Gulu City and Gulu District) are situated, has a low cattle population, what then is the underlying rationale for selecting this area for the study? | We thank the reviewer for this important observation. We have revised Line 100-101  See L143 and L144 |
| 15 | How were the authors able to just target 380 farmers? How and where did you obtain your population sample size. This should be described. Also, present the sample size table as written in Line 110 | Thank for this observation. We relied on official records obtained from the District/City veterinary officer. The focus was on the number of registered smallholder dairy farmers in the selected subcounties. The multistage approach allowed for the selection of the study participants.  See L128-136 |
| 16 | “divisions and sub-counties were stratified”. How many divisions and sub-counties were selected? | Thank you for this observation. In this study a multi-stage sampling technique was applied to select three sub-counties from Gulu District (Awach, Paicho, and Patiko) and two divisions from Gulu City (Laroo-Pece and Bardege-Layibi) which were randomly selected from the 11 sub-counties and four divisions, respectively.  See L170-176 |
|  | **Editor** |  |
| 1 | Please ensure that your manuscript meets PLOS ONE's style requirements, including those for file naming. | Thank you this information we have revised and formatted the manuscript according to PLOS ONE’s style requirements. |
| 2 | Please state what role the funders took in the study. If the funders had no role, please state: "The funders had no role in study design, data collection and analysis, decision to publish, or preparation of the manuscript." | The funders had no role in study design, data collection and analysis, decision to publish, or preparation of the manuscript |
| 3 | If there are ethical or legal restrictions on sharing a de-identified data set, please explain them in detail (e.g., data contain potentially identifying or sensitive patient information, data are owned by a third-party organization, etc.) and who has imposed them (e.g., a Research Ethics Committee or Institutional Review Board, etc.). Please also provide contact information for a data access committee, ethics committee, or other institutional body to which data requests may be sent. | There is no ethical or legal restrictions on sharing the data |
|  | If there are no restrictions, please upload the minimal anonymized data set necessary to replicate your study findings to a stable, public repository and provide us with the relevant URLs, DOIs, or accession numbers. | This will be uploaded soon |
|  | Please update your Data Availability statement in the submission form accordingly. | Thank you, we have revised and updated the data availability statement. |
