# Supplementary material for: Profit efficiency of smallholder dairy production systems: Empirical evidence from Northern Uganda
Source: PLoS One. 2026 Aug 24;21(8):e0333337. doi: 10.1371/journal.pone.0333337 (PMC13502703; doi:10.1371/journal.pone.0333337)
Supplement: Supplementary file 3 [file pone.0333337.s003.docx]

**Comments to Authors**

Many thanks to the authors for addressing all raised concerns. I have some other concerns that should be addressed to further strengthen the manuscript

1. Line 112, Authors should be careful of making absolute statements like this “Most existing studies have focused on milk production…,”, they should rather state “Most existing studies known to the authors have focused on milk production…,
2. Thank you for including the map. However, Gulu district and Gulu City should be shown as well in the map together with their sub-counties and divisions. Kindly make necessary adjustments
3. Authors should either show the Krejcie and Morgan table or write out a formular of how the sample size 191 was achieved
4. What is this? “Error! Not a valid bookmark self-reference.” Line 448 (Section 3.6)
