# Supplementary material for: Profit efficiency of smallholder dairy production systems: Empirical evidence from Northern Uganda
Source: PLoS One. 2026 Aug 24;21(8):e0333337. doi: 10.1371/journal.pone.0333337 (PMC13502703; doi:10.1371/journal.pone.0333337)
Supplement: Supplementary file 4 [file pone.0333337.s004.docx]

**Point-by-point response to reviewers and editors**

**Date:** 01/July/2026

**Ref: Submission ID:** PONE-D-25-49874

**Title of Article:** Profit Efficiency of Smallholder Dairy Production Systems: Empirical evidence from northern Uganda

Dear Editor,

Dear Reviewers,

We are pleased to respond to the editorial and review comments raised. The revised manuscript submitted together with this response letter, has all changes highlighted in yellow.

Below is a detailed explanation of how each of the issues raise have been addressed.

| **S/N** | **Issue** | **Response** |
| --- | --- | --- |
|  | **Reviewer #2:** |  |
| 1 | Line 112, Authors should be careful of making absolute statements like this “Most existing studies have focused on milk production…,”, they should rather state “Most existing studies known to the authors have focused on milk production…, | We thank the reviewer for this important observation. We have revised and corrected on the use of absolute statements  See L 112-113 |
| 2 | Thank you for including the map. However, Gulu district and Gulu City should be shown as well in the map together with their sub-counties and divisions. Kindly make necessary adjustments. | Thank you for the feedback, we have revised and corrected the map of the study area  See Figure 2 |
| 3 | Authors should either show the Krejcie and Morgan table or write out a formular of how the sample size 191 was achieved | Thank you for pointing this out. We have revised and included Krejcie and Morgan’s formular for sample size determination as indicated in equation one  See L 165-175 |
| 4 | What is this? “Error! Not a valid bookmark self-reference.” Line 448 (Section 3.6) | We thank the reviewer for this important observation. We have revised and removed the Error! Not a valid bookmark self-reference  See L449 |
