# Supplementary material for: Profit efficiency of smallholder dairy production systems: Empirical evidence from Northern Uganda
Source: PLoS One. 2026 Aug 24;21(8):e0333337. doi: 10.1371/journal.pone.0333337 (PMC13502703; doi:10.1371/journal.pone.0333337)
Supplement: Peer review history

# Peer review: Decision Letter 0

**DOI:** 10.1371/journal.pone.0333337.r001

**Authors:** Olutosin Ademola Otekunrin

7 May 2026

Dear Dr. Oyoo,

Thank you for submitting your manuscript to PLOS ONE. After careful consideration, we feel that it has merit but does not fully meet PLOS ONE’s publication criteria as it currently stands. Therefore, we invite you to submit a revised version of the manuscript that addresses the points raised during the review process.

==============================

Please submit your revised manuscript by Jun 21 2026 11:59PM. If you will need more time than this to complete your revisions, please reply to this message or contact the journal office at plosone@plos.org. When you're ready to submit your revision, log on to https://www.editorialmanager.com/pone/ and select the 'Submissions Needing Revision' folder to locate your manuscript file.

- A letter that responds to each point raised by the academic editor and reviewer(s). You should upload this letter as a separate file labeled 'Response to Reviewers'.
- A marked-up copy of your manuscript that highlights changes made to the original version. You should upload this as a separate file labeled 'Revised Manuscript with Track Changes'.
- An unmarked version of your revised paper without tracked changes. You should upload this as a separate file labeled 'Manuscript'.

If you would like to make changes to your financial disclosure, please include your updated statement in your cover letter. Guidelines for resubmitting your figure files are available below the reviewer comments at the end of this letter.

If applicable, we recommend that you deposit your laboratory protocols in protocols.io to enhance the reproducibility of your results. Protocols.io assigns your protocol its own identifier (DOI) so that it can be cited independently in the future. For instructions see: https://journals.plos.org/plosone/s/submission-guidelines#loc-laboratory-protocols. Additionally, PLOS ONE offers an option for publishing peer-reviewed Lab Protocol articles, which describe protocols hosted on protocols.io. Read more information on sharing protocols at https://plos.org/protocols?utm_medium=editorial-email&utm_source=authorletters&utm_campaign=protocols.

As the corresponding author, your ORCID iD is verified in the submission system and will appear in the published article. PLOS supports the use of ORCID, and we encourage all coauthors to register for an ORCID iD and use it as well. Please encourage your coauthors to verify their ORCID iD within the submission system before final acceptance, as unverified ORCID iDs will not appear in the published article. *Only*  the individual author can complete the verification step; PLOS staff *cannot*  verify ORCID iDs on behalf of authors.

We look forward to receiving your revised manuscript.

Kind regards,

Olutosin Ademola Otekunrin, PhD

Academic Editor

PLOS One

Journal requirements:

When submitting your revision, we need you to address these additional requirements.

1. Please ensure that your manuscript meets PLOS ONE's style requirements, including those for file naming. The PLOS ONE style templates can be found at

https://journals.plos.org/plosone/s/file?id=wjVg/PLOSOne_formatting_sample_main_body.pdf and

https://journals.plos.org/plosone/s/file?id=ba62/PLOSOne_formatting_sample_title_authors_affiliations.pdf

2. Thank you for stating the following financial disclosure:

“The study was funded by The Regional Universities Forum for Capacity Building in Agriculture (RUFORUM).”

Please state what role the funders took in the study.  If the funders had no role, please state: "The funders had no role in study design, data collection and analysis, decision to publish, or preparation of the manuscript."

If this statement is not correct you must amend it as needed.

Please include this amended Role of Funder statement in your cover letter; we will change the online submission form on your behalf.

3. Thank you for stating the following in the Funding Section of your manuscript:

“The author received funding from Regional Universities Forum for Capacity Building in Agriculture (RUFORUM) (TAGDev 101)”

We note that you have provided funding information that is currently declared in your Funding Statement. However, funding information should not appear in the Acknowledgments section or other areas of your manuscript. We will only publish funding information present in the Funding Statement section of the online submission form.

Please remove any funding-related text from the manuscript and let us know how you would like to update your Funding Statement. Currently, your Funding Statement reads as follows:

“The study was funded by The Regional Universities Forum for Capacity Building in Agriculture (RUFORUM).”

Please include your amended statements within your cover letter; we will change the online submission form on your behalf.

4. We note that you have indicated that there are restrictions to data sharing for this study. For studies involving human research participant data or other sensitive data, we encourage authors to share de-identified or anonymized data. However, when data cannot be publicly shared for ethical reasons, we allow authors to make their data sets available upon request. For information on unacceptable data access restrictions, please see http://journals.plos.org/plosone/s/data-availability#loc-unacceptable-data-access-restrictions.

Before we proceed with your manuscript, please address the following prompts:

a) If there are ethical or legal restrictions on sharing a de-identified data set, please explain them in detail (e.g., data contain potentially identifying or sensitive patient information, data are owned by a third-party organization, etc.) and who has imposed them (e.g., a Research Ethics Committee or Institutional Review Board, etc.). Please also provide contact information for a data access committee, ethics committee, or other institutional body to which data requests may be sent.

b) If there are no restrictions, please upload the minimal anonymized data set necessary to replicate your study findings to a stable, public repository and provide us with the relevant URLs, DOIs, or accession numbers. Please see http://www.bmj.com/content/340/bmj.c181.long for guidelines on how to de-identify and prepare clinical data for publication. For a list of recommended repositories, please see https://journals.plos.org/plosone/s/recommended-repositories. You also have the option of uploading the data as Supporting Information files, but we would recommend depositing data directly to a data repository if possible.

Please update your Data Availability statement in the submission form accordingly.

If the reviewer comments include a recommendation to cite specific previously published works, please review and evaluate these publications to determine whether they are relevant and should be cited. There is no requirement to cite these works unless the editor has indicated otherwise.

Additional Editor Comments:

Experts in the field have reviewed you manuscript and you are expected to address their comments as early as possible. Thank you.

[Note: HTML markup is below. Please do not edit.]

Reviewers' comments:

Reviewer's Responses to Questions

Comments to the Author

1. Is the manuscript technically sound, and do the data support the conclusions?

The manuscript must describe a technically sound piece of scientific research with data that supports the conclusions. Experiments must have been conducted rigorously, with appropriate controls, replication, and sample sizes. The conclusions must be drawn appropriately based on the data presented.

Reviewer #1: No

Reviewer #2: Yes

**********

2. Has the statistical analysis been performed appropriately and rigorously?

Reviewer #1: No

Reviewer #2: Yes

**********

3. Have the authors made all data underlying the findings in their manuscript fully available?

The PLOS Data policy requires authors to make all data underlying the findings described in their manuscript fully available without restriction, with rare exception (please refer to the Data Availability Statement in the manuscript PDF file). The data should be provided as part of the manuscript or its supporting information, or deposited to a public repository. For example, in addition to summary statistics, the data points behind means, medians and variance measures should be available. If there are restrictions on publicly sharing data—e.g. participant privacy or use of data from a third party—those must be specified.

Reviewer #1: No

Reviewer #2: Yes

**********

4. Is the manuscript presented in an intelligible fashion and written in standard English?

PLOS ONE does not copyedit accepted manuscripts, so the language in submitted articles must be clear, correct, and unambiguous. Any typographical or grammatical errors should be corrected at revision, so please note any specific errors here.

Reviewer #1: No

Reviewer #2: Yes

**********

5. Review Comments to the Author

Please use the space provided to explain your answers to the questions above. You may also include additional comments for the author, including concerns about dual publication, research ethics, or publication ethics. (Please upload your review as an attachment if it exceeds 20,000 characters)

Reviewer #1: I found several methodological issues in the paper:

Cobb-Douglas was used, but no justification or testing provided. It has some restrictive assumptions and may not hold across heterogeneous smallholder systems. A comparitive test should be conducted.

Gamma refers to what the gap between a farmer and the frontier is due to genuine inefficiency versus random noise (weather, measurement error, luck). Their gamma is 0.038 — meaning only 4% of that gap is inefficiency and 96% is just noise. Thus, there's almost no systematic inefficiency which means farmers are mostly affected by random factors. But authors are analysing what causes inefficiency — age, education, credit access, etc. Either acknowledge inefficiency, or explain why the analysis is still valid despite the low gamma.

Zero-grazing is presented in the paper as the better system due to better management, higher gross margins, etc., but in Table 7 it shows that zero-grazing farmers have the worst average efficiency score, while herding and tethered farmers score higher. This is not what the discussion claims.

For each production system, they estimate a separate model with around 15 parameters. The herding group has only 47 farmers. You need at least 10 observations per parameter for stable estimates. With 47 farmers and 15 parameters, the estimates become unreliable.

The authors list many (20+) variables and test them, but no reasoning is provided to motivate why these variables matter for profit efficiency. Apply a conceptual framework to provide a motivation for including certain variables.

Reviewer #2: Comments to Authors

The manuscript presents an important comparative analysis of profit efficiency across three dairy production systems. However, there are several concerns regarding the overall strength of the manuscript.

1.Can the Authors provide a map that clearly represents the study area?

2.Authors should recast lines 216 and 217. They may wish to adopt something like “Table 2 describes the…”

3.I can see that authors have more or less carried out a comparative study across three dairy practitioners: zero-grazing, Tethered, and herding. However, this very important consideration was not included in the abstract. Authors are therefore advised to consider rewriting the abstract to capture this. In addition, there should be a specific mention of results, especially figures/metrics.

4.Where is the formula adopted for the sample size selection?

5.Lines 117,118,119 – authors should recast to correct ambiguity. Could be restructured like this: A face-to-face interview was done using a structured questionnaire.

6.A lot of work has been done on all the types of efficiencies mentioned in the manuscript. Allocative, technical, and economic efficiencies. Also, SFA. Authors should cite and support old references with recent ones.

7.Authors should recast lines 228 and 229

8.Authors should endeavour to arrange their references in ascending order by year and not by alphabets

9.As much as possible, Authors should add the DOIs to references

10.In Table 4, the Equipment cost is not significant, Authors should endeavour to remove the asterisk

11.In section 2.3, the authors should update references with more recent ones; the references are too old. Other sections should be reviewed as well.

12.Line 136- Authors may wish to swap “serious statistical analysis” with “rigorous statistical analysis.”

13.Where are the diagnostic test results of the Chi-Square and the ANOVA? Authors wrote- “… and a one-ANOVA test.” Do you mean a one-way ANOVA? If your Chi-Square result is Table 3, please label it as such and describe the same in your write-up as well.

14.Lines 100-101 - If the Acholi region, where the study locations (Gulu City and Gulu District) are situated, has a low cattle population, what then is the underlying rationale for selecting this area for the study?

15.How were the authors able to just target 380 farmers? How and where did you obtain your population sample size. This should be described. Also, present the sample size table as written in Line 110

16.“divisions and sub-counties were stratified”. How many divisions and sub-counties were selected?

**********

6. PLOS authors have the option to publish the peer review history of their article (what does this mean?). If published, this will include your full peer review and any attached files.

If you choose “no”, your identity will remain anonymous but your review may still be made public.

Do you want your identity to be public for this peer review? For information about this choice, including consent withdrawal, please see our Privacy Policy.

Reviewer #1: No

Reviewer #2: No

**********

[NOTE: If reviewer comments were submitted as an attachment file, they will be attached to this email and accessible via the submission site. Please log into your account, locate the manuscript record, and check for the action link "View Attachments". If this link does not appear, there are no attachment files.]

To ensure your figures meet our technical requirements, please review our figure guidelines: https://journals.plos.org/plosone/s/figures

You may also use PLOS’s free figure tool, NAAS, to help you prepare publication quality figures: https://journals.plos.org/plosone/s/figures#loc-tools-for-figure-preparation.

NAAS will assess whether your figures meet our technical requirements by comparing each figure against our figure specifications.

Attachment Submitted filename: Comments_profit efficiency.docx

# Author comment: Author response to Decision Letter 1

**DOI:** 10.1371/journal.pone.0333337.r002

7 Jun 2026

S/N Issue Response

Reviewer #1:

1 Cobb-Douglas was used, but no justification or testing provided. It has some restrictive assumptions and may not hold across heterogeneous smallholder systems. A comparative test should be conducted. Thank you for pointing this out. We have revised and included the justification for the use of Cobb-Douglas and comparative test was included.

To identify the most appropriate functional form for estimating profit efficiency among smallholder dairy farmers, both the Cobb-Douglas and Translog stochastic frontier specifications were estimated and systematically compared using established econometric and statistical criteria. The results are presented in However, when disaggregated by production system, the results reveal a more nuanced picture. For specific dairy production systems, the Cobb-Douglas specification consistently outperformed the Translog model.

Thus, it’s the results of the Cobb-Douglas model that are presented

See L295 – 214 and Table 3.

2 Gamma refers to what the gap between a farmer and the frontier is due to genuine inefficiency versus random noise (weather, measurement error, luck). Their gamma is 0.038 — meaning only 4% of that gap is inefficiency and 96% is just noise. Thus, there's almost no systematic inefficiency which means farmers are mostly affected by random factors. But authors are analysing what causes inefficiency — age, education, credit access, etc. Either acknowledge inefficiency or explain why the analysis is still valid despite the low gamma. Thank you for this observation.

We have revised our analysis and corrected this error.

See L441-448 and Table 8.

3 Zero-grazing is presented in the paper as the better system due to better management, higher gross margins, etc., but in Table 7 it shows that zero-grazing farmers have the worst average efficiency score, while herding and tethered farmers score higher. This is not what the discussion claims. We thank the reviewer for this important observation. We have revised and corrected the efficiency scores

See Table 9

4 For each production system, they estimate a separate model with around 15 parameters. The herding group has only 47 farmers. You need at least 10 observations per parameter for stable estimates. With 47 farmers and 15 parameters, the estimates become unreliable. We thank the reviewer for this important observation. We have revised and removed other variables

See Table 7

5 The authors list many (20+) variables and test them, but no reasoning is provided to motivate why these variables matter for profit efficiency. Apply a conceptual framework to provide a motivation for including certain variables. We thank the reviewer for this important observation. We have revised and included the conceptual framework to provide the motivation for including certain variables

See Figure 1

Reviewer #2:

1 Can the Authors provide a map that clearly represents the study area? Thank you for the feedback, we have revised and included and included the map of the study area

2 Authors should recast lines 216 and 217. They may wish to adopt something like “Table 2 describes the…” We thank the reviewer for this valuable suggestion; we have revised and recasted lines 216 and 217

See L328 and L329

3 I can see that authors have more or less carried out a comparative study across three dairy practitioners: zero-grazing, Tethered, and herding. However, this very important consideration was not included in the abstract. Authors are therefore advised to consider rewriting the abstract to capture this. In addition, there should be a specific mention of results, especially figures/metrics.

Thank you for the feedback, we have revised and included and included the results especially figures in the abstract

See Abstract

4 Where is the formula adopted for the sample size selection? Thank for this observation. The sample size for this followed the Krejcie & Morgan (1970) table of sample size determination. This is presented in L128 - 138

5 Lines 117,118,119 – authors should recast to correct ambiguity. Could be restructured like this: A face-to-face interview was done using a structured questionnaire. We thank the reviewer for this valuable suggestion; we have revised and recasted lines 117, 118, and 119

See L179, L180, and L181

6 A lot of work has been done on all the types of efficiencies mentioned in the manuscript. Allocative, technical, and economic efficiencies. Also, SFA. Authors should cite and support old references with recent ones. We thank the reviewer for this important observation. We have cited and supported the old references with recent ones

7 Authors should recast lines 228 and 229 We thank the reviewer for this valuable suggestion; we have revised and recasted lines 228 and 229

See L339 and L340

8 Authors should endeavour to arrange their references in ascending order by year and not by alphabets We thank the reviewer for this important observation. We have arranged the references in ascending order by year and not by alphabets

9 As much as possible, Authors should add the DOIs to references We thank the reviewer for this important observation. We have added the DOIs to the references

10 In Table 4, the Equipment cost is not significant, Authors should endeavour to remove the asterisk We have removed the asterisk

See Table 6

11 In section 2.3, the authors should update references with more recent ones; the references are too old. Other sections should be reviewed as well. We thank the reviewer for this observation and for highlighting the need for inclusion of recent references. We have revised and included the recent references

12 Line 136- Authors may wish to swap “serious statistical analysis” with “rigorous statistical analysis.” We thank the review for this suggestion. We have revised and swapped “serious” with rigorous

See L198

13 Where are the diagnostic test results of the Chi-Square and the ANOVA? Authors wrote- “… and a one-ANOVA test.” Do you mean a one-way ANOVA? If your Chi-Square result is Table 3, please label it as such and describe the same in your write-up as well. We thank the reviewer for this important observation. We have revised and one-way ANOVA and Chi-square result in the table and describe the same in the write-up

See L328, L329, L335, L336, L339, L340, L346, and L347

14 Lines 100-101 - If the Acholi region, where the study locations (Gulu City and Gulu District) are situated, has a low cattle population, what then is the underlying rationale for selecting this area for the study? We thank the reviewer for this important observation. We have revised Line 100-101

See L143 and L144

15 How were the authors able to just target 380 farmers? How and where did you obtain your population sample size. This should be described. Also, present the sample size table as written in Line 110 Thank for this observation. We relied on official records obtained from the District/City veterinary officer. The focus was on the number of registered smallholder dairy farmers in the selected subcounties. The multistage approach allowed for the selection of the study participants.

See L128-136

16 “divisions and sub-counties were stratified”. How many divisions and sub-counties were selected? Thank you for this observation. In this study a multi-stage sampling technique was applied to select three sub-counties from Gulu District (Awach, Paicho, and Patiko) and two divisions from Gulu City (Laroo-Pece and Bardege-Layibi) which were randomly selected from the 11 sub-counties and four divisions, respectively.

See L170-176

Editor

1 Please ensure that your manuscript meets PLOS ONE's style requirements, including those for file naming. Thank you this information we have revised and formatted the manuscript according to PLOS ONE’s style requirements.

2 Please state what role the funders took in the study. If the funders had no role, please state: "The funders had no role in study design, data collection and analysis, decision to publish, or preparation of the manuscript." The funders had no role in study design, data collection and analysis, decision to publish, or preparation of the manuscript

3 If there are ethical or legal restrictions on sharing a de-identified data set, please explain them in detail (e.g., data contain potentially identifying or sensitive patient information, data are owned by a third-party organization, etc.) and who has imposed them (e.g., a Research Ethics Committee or Institutional Review Board, etc.). Please also provide contact information for a data access committee, ethics committee, or other institutional body to which data requests may be sent. There is no ethical or legal restrictions on sharing the data

If there are no restrictions, please upload the minimal anonymized data set necessary to replicate your study findings to a stable, public repository and provide us with the relevant URLs, DOIs, or accession numbers. This will be uploaded soon

Please update your Data Availability statement in the submission form accordingly. Thank you, we have revised and updated the data availability statement.

Attachment Submitted filename: Responses to review comments R1.docx

# Peer review: Decision Letter 1

**DOI:** 10.1371/journal.pone.0333337.r003

**Authors:** Olutosin Ademola Otekunrin

24 Jun 2026

Dear Dr. Oyoo,

Thank you for submitting your manuscript to PLOS ONE. After careful consideration, we feel that it has merit but does not fully meet PLOS ONE’s publication criteria as it currently stands. Therefore, we invite you to submit a revised version of the manuscript that addresses the points raised during the review process.

**ACADEMIC EDITOR:**  Kindly address the comments of the reviewer before this manuscript can move to the next stage. Thank you.

Please submit your revised manuscript by Aug 08 2026 11:59PM. If you will need more time than this to complete your revisions, please reply to this message or contact the journal office at plosone@plos.org. When you're ready to submit your revision, log on to https://www.editorialmanager.com/pone/ and select the 'Submissions Needing Revision' folder to locate your manuscript file.

- A letter that responds to each point raised by the academic editor and reviewer(s). You should upload this letter as a separate file labeled 'Response to Reviewers'.
- A marked-up copy of your manuscript that highlights changes made to the original version. You should upload this as a separate file labeled 'Revised Manuscript with Track Changes'.
- An unmarked version of your revised paper without tracked changes. You should upload this as a separate file labeled 'Manuscript'.

If you would like to make changes to your financial disclosure, please include your updated statement in your cover letter. Guidelines for resubmitting your figure files are available below the reviewer comments at the end of this letter.

If applicable, we recommend that you deposit your laboratory protocols in protocols.io to enhance the reproducibility of your results. Protocols.io assigns your protocol its own identifier (DOI) so that it can be cited independently in the future. For instructions see: https://journals.plos.org/plosone/s/submission-guidelines#loc-laboratory-protocols. Additionally, PLOS ONE offers an option for publishing peer-reviewed Lab Protocol articles, which describe protocols hosted on protocols.io. Read more information on sharing protocols at https://plos.org/protocols?utm_medium=editorial-email&utm_source=authorletters&utm_campaign=protocols.

As the corresponding author, your ORCID iD is verified in the submission system and will appear in the published article. PLOS supports the use of ORCID, and we encourage all coauthors to register for an ORCID iD and use it as well. Please encourage your coauthors to verify their ORCID iD within the submission system before final acceptance, as unverified ORCID iDs will not appear in the published article. *Only*  the individual author can complete the verification step; PLOS staff *cannot*  verify ORCID iDs on behalf of authors.

We look forward to receiving your revised manuscript.

Kind regards,

Olutosin Ademola Otekunrin, PhD

Academic Editor

PLOS One

**Journal Requirements:**

1. If the reviewer comments include a recommendation to cite specific previously published works, please review and evaluate these publications to determine whether they are relevant and should be cited. There is no requirement to cite these works unless the editor has indicated otherwise.

2. Please review your reference list to ensure that it is complete and correct. If you have cited papers that have been retracted, please include the rationale for doing so in the manuscript text, or remove these references and replace them with relevant current references. Any changes to the reference list should be mentioned in the rebuttal letter that accompanies your revised manuscript. If you need to cite a retracted article, indicate the article’s retracted status in the References list and also include a citation and full reference for the retraction notice.

[Note: HTML markup is below. Please do not edit.]

Reviewers' comments:

Reviewer's Responses to Questions

Reviewer #2: (No Response)

**********

2. Is the manuscript technically sound, and do the data support the conclusions?

Reviewer #2: Yes

**********

3. Has the statistical analysis been performed appropriately and rigorously?

Reviewer #2: Yes

**********

4. Have the authors made all data underlying the findings in their manuscript fully available?

The PLOS Data policy

Reviewer #2: Yes

**********

5. Is the manuscript presented in an intelligible fashion and written in standard English?

Reviewer #2: Yes

**********

Reviewer #2: Comments to Authors

Many thanks to the authors for addressing all raised concerns. I have some other concerns that should be addressed to further strengthen the manuscript

1.Line 112, Authors should be careful of making absolute statements like this “Most existing studies have focused on milk production…,”, they should rather state “Most existing studies known to the authors have focused on milk production…,

2.Thank you for including the map. However, Gulu district and Gulu City should be shown as well in the map together with their sub-counties and divisions. Kindly make necessary adjustments

3.Authors should either show the Krejcie and Morgan table or write out a formular of how the sample size 191 was achieved

4.What is this? “Error! Not a valid bookmark self-reference.” Line 448 (Section 3.6)

**********

what does this mean?). If published, this will include your full peer review and any attached files.

If you choose “no”, your identity will remain anonymous but your review may still be made public.

Do you want your identity to be public for this peer review? For information about this choice, including consent withdrawal, please see our Privacy Policy

Reviewer #2: No

**********

[NOTE: If reviewer comments were submitted as an attachment file, they will be attached to this email and accessible via the submission site. Please log into your account, locate the manuscript record, and check for the action link "View Attachments". If this link does not appear, there are no attachment files.]

To ensure your figures meet our technical requirements, please review our figure guidelines: https://journals.plos.org/plosone/s/figures

You may also use PLOS’s free figure tool, NAAS, to help you prepare publication quality figures: https://journals.plos.org/plosone/s/figures#loc-tools-for-figure-preparation.

NAAS will assess whether your figures meet our technical requirements by comparing each figure against our figure specifications.

Attachment Submitted filename: Comments to Authors_Dairy2.docx

# Author comment: Author response to Decision Letter 2

**DOI:** 10.1371/journal.pone.0333337.r004

1 Jul 2026

Point-by-point response to reviewers and editors

Date: 01/July/2026

Ref: Submission ID: PONE-D-25-49874

Title of Article: Profit Efficiency of Smallholder Dairy Production Systems: Empirical evidence from northern Uganda

Dear Editor,

Dear Reviewers,

We are pleased to respond to the editorial and review comments raised. The revised manuscript submitted together with this response letter, has all changes highlighted in yellow.

Below is a detailed explanation of how each of the issues raise have been addressed.

S/N Issue Response

Reviewer #2:

1 Line 112, Authors should be careful of making absolute statements like this “Most existing studies have focused on milk production…,”, they should rather state “Most existing studies known to the authors have focused on milk production…, We thank the reviewer for this important observation. We have revised and corrected on the use of absolute statements

See L 112-113

2 Thank you for including the map. However, Gulu district and Gulu City should be shown as well in the map together with their sub-counties and divisions. Kindly make necessary adjustments. Thank you for the feedback, we have revised and corrected the map of the study area

See Figure 2

3 Authors should either show the Krejcie and Morgan table or write out a formular of how the sample size 191 was achieved Thank you for pointing this out. We have revised and included Krejcie and Morgan’s formular for sample size determination as indicated in equation one

See L 165-175

4 What is this? “Error! Not a valid bookmark self-reference.” Line 448 (Section 3.6) We thank the reviewer for this important observation. We have revised and removed the Error! Not a valid bookmark self-reference

See L449

Attachment Submitted filename: Responses to review comments R2.docx

# Peer review: Decision Letter 2

**DOI:** 10.1371/journal.pone.0333337.r005

**Authors:** Olutosin Ademola Otekunrin

22 Jul 2026

Profit Efficiency of Smallholder Dairy Production Systems: Empirical evidence from northern Uganda

PONE-D-25-49874R2

Dear Dr. Oyoo,

We’re pleased to inform you that your manuscript has been judged scientifically suitable for publication and will be formally accepted for publication once it meets all outstanding technical requirements.

Within one week, you’ll receive an e-mail detailing the required amendments. When these have been addressed, you’ll receive a formal acceptance letter and your manuscript will be scheduled for publication.

An invoice will be generated when your article is formally accepted. Please note, if your institution has a publishing partnership with PLOS and your article meets the relevant criteria, all or part of your publication costs will be covered. Please make sure your user information is up-to-date by logging into Editorial Manager at Editorial Manager® and clicking the ‘Update My Information' link at the top of the page. For questions related to billing, please contact billing support.

If your institution or institutions have a press office, please notify them about your upcoming paper to help maximize its impact. If they’ll be preparing press materials, please inform our press team as soon as possible -- no later than 48 hours after receiving the formal acceptance. Your manuscript will remain under strict press embargo until 2 pm Eastern Time on the date of publication. For more information, please contact onepress@plos.org.

Kind regards,

Olutosin Ademola Otekunrin, PhD

Academic Editor

PLOS One

Additional Editor Comments (optional):

Reviewers' comments:

Reviewer's Responses to Questions

**Comments to the Author**

Reviewer #2: All comments have been addressed

**********

2. Is the manuscript technically sound, and do the data support the conclusions?

Reviewer #2: Yes

**********

3. Has the statistical analysis been performed appropriately and rigorously?

Reviewer #2: Yes

**********

4. Have the authors made all data underlying the findings in their manuscript fully available?

The PLOS Data policy

Reviewer #2: Yes

**********

5. Is the manuscript presented in an intelligible fashion and written in standard English?

Reviewer #2: Yes

**********

Reviewer #2: Many thanks to the authors for making considerable efforts to address the issues raised on their manuscript.

**********

what does this mean?). If published, this will include your full peer review and any attached files.

If you choose “no”, your identity will remain anonymous but your review may still be made public.

**Do you want your identity to be public for this peer review?**  For information about this choice, including consent withdrawal, please see our Privacy Policy

Reviewer #2: No

**********

# Editor report: Acceptance letter

**DOI:** 10.1371/journal.pone.0333337.r006

**Authors:** Olutosin Ademola Otekunrin

PONE-D-25-49874R2

PLOS One

Dear Dr. Oyoo,

I'm pleased to inform you that your manuscript has been deemed suitable for publication in PLOS One. Congratulations! Your manuscript is now being handed over to our production team.

At this stage, our production department will prepare your paper for publication. This includes ensuring the following:

* All references, tables, and figures are properly cited

* All relevant supporting information is included in the manuscript submission,

* There are no issues that prevent the paper from being properly typeset

You will receive further instructions from the production team, including instructions on how to review your proof when it is ready. Please keep in mind that we are working through a large volume of accepted articles, so please give us a few days to review your paper and let you know the next and final steps.

Lastly, if your institution or institutions have a press office, please let them know about your upcoming paper now to help maximize its impact. If they'll be preparing press materials, please inform our press team within the next 48 hours. Your manuscript will remain under strict press embargo until 2 pm Eastern Time on the date of publication. For more information, please contact onepress@plos.org.

You will receive an invoice from PLOS for your publication fee after your manuscript has reached the completed accept phase. If you receive an email requesting payment before acceptance or for any other service, this may be a phishing scheme. Learn how to identify phishing emails and protect your accounts at https://explore.plos.org/phishing.

If we can help with anything else, please email us at customercare@plos.org.

Thank you for submitting your work to PLOS One and supporting open access.

Kind regards,

PLOS ONE Editorial Office Staff

on behalf of

Dr. Olutosin Ademola Otekunrin

Academic Editor

PLOS One
